# An in silico method to assess antibody fragment polyreactivity

**DOI:** 10.1038/s41467-022-35276-4

**Published:** 2022-12-07

**Authors:** Edward P. Harvey, Jung-Eun Shin, Meredith A. Skiba, Genevieve R. Nemeth, Joseph D. Hurley, Alon Wellner, Ada Y. Shaw, Victor G. Miranda, Joseph K. Min, Chang C. Liu, Debora S. Marks, Andrew C. Kruse

**Affiliations:** 1 Department of Biological Chemistry and Molecular Pharmacology, Blavatnik Institute, Harvard Medical School, Boston, MA 02115 USA; 2 Department of Systems Biology, Harvard Medical School, Boston, MA 02115 USA; 3 Department of Chemistry, University of California, Irvine, CA 92697 USA; 4 Department of Molecular Biology & Biochemistry, University of California, Irvine, CA 92697 USA; 5 Department of Biomedical Engineering, University of California, Irvine, CA 92692 USA; 6 Broad Institute of Harvard and MIT, Cambridge, MA 02142 USA

**Keywords:** Biologics, Machine learning, X-ray crystallography

## Abstract

Antibodies are essential biological research tools and important therapeutic agents, but some exhibit non-specific binding to off-target proteins and other biomolecules. Such polyreactive antibodies compromise screening pipelines, lead to incorrect and irreproducible experimental results, and are generally intractable for clinical development. Here, we design a set of experiments using a diverse naïve synthetic camelid antibody fragment (nanobody) library to enable machine learning models to accurately assess polyreactivity from protein sequence (AUC > 0.8). Moreover, our models provide quantitative scoring metrics that predict the effect of amino acid substitutions on polyreactivity. We experimentally test our models’ performance on three independent nanobody scaffolds, where over 90% of predicted substitutions successfully reduced polyreactivity. Importantly, the models allow us to diminish the polyreactivity of an angiotensin II type I receptor antagonist nanobody, without compromising its functional properties. We provide a companion web-server that offers a straightforward means of predicting polyreactivity and polyreactivity-reducing mutations for any given nanobody sequence.

## Abstract (web summary)

Off-target binding hinders the development of therapeutic antibodies and reproducibility in basic research settings. Here the authors develop a method to quantify and reduce the polyreactivity of antibody fragments based on protein sequence alone.

## Introduction

Due to their specificity and affinity, antibodies are an indispensable class of biomedical research tools as well as important therapeutics for the treatment of cancer, autoimmune, and infectious diseases. Although high target specificity is prioritized during the antibody discovery process, some antibodies with desired functional properties bind to off-target biomolecules with low-affinity. In clinical development, these non-specific or polyreactive antibodies show poor pharmacokinetics or other liabilities that limit clinical use^1–3^. Additionally, polyreactive antibodies encountered in the basic research setting cause misinterpretation of results, low reproducibility in routine experiments, and wasted time and money^4^. Thus, there have been several calls to standardize the quality and specificity of antibodies used in research settings similar to those in the clinic^5,6^.

Synthetic antibody libraries facilitate antibody discovery for targets that are not readily amenable to traditional immunization-based selection campaigns, such as those that are highly conserved across species^7–13^. However, antibodies discovered through fully synthetic approaches lack in vivo filtering for off-target reactivity. Developing and improving methods to detect and quantify polyreactivity are essential for improving our ability to obtain high quality antibodies through fully synthetic means and enhancing the quality of antibodies in both clinical development and basic research settings. Many experimental methods that evaluate polyreactivity^14–21^ are low-throughput and require experimental screening with purified antibody. The degree of polyreactivity is highly method and reagent-dependent and is often measured after antigen selection once lead clones are already identified. Understanding sequence features of polyreactive antibodies could provide an efficient avenue to quantitatively assess antibody polyreactivity based on a standard data set and prioritize clones with the highest clinical and research potential. Previous studies^22–29^ have revealed determinants of polyreactivity in antibodies, such as specific J- and V-chain usage^24^, high isoelectric points^23,25–32^, longer CDR3s^23,30^, enrichment of arginine, glycine, valine, and tryptophan containing motifs^25^, and glutamine residues^30^. Despite these extensive analyses, the relative importance of many characteristics is disputed^28^ and currently available software cannot predict polyreactivity quantitatively^24^.

For broad utility, a computational method should accurately predict the *degree* of polyreactivity of diverse sequences and generate candidate rescue mutations from sequence alone. To achieve this goal, we designed experiments to learn features of high and low polyreactivity clones from a naïve synthetic yeast display library of heavy-chain only camelid antibody fragments (nanobodies)^7,33^. Synthetic nanobodies provide an ideal reductionist system to probe polyreactivity in the context of a fixed framework without the influence of heavy and light chain pairing effects. Furthermore, nanobodies are emerging therapeutic molecules that can target antigen surfaces and tissue types not accessible to conventional antibodies^34,35^. One nanobody is approved for clinical use and increasing numbers are advancing through clinical stages^35,36^. Despite growing interest in nanobodies as therapeutic tools, few developability studies focus on single chain antibody fragments.

Here, we show that learned features of high and low polyreactivity nanobodies result in generalizable software that quantifies nanobody polyreactivity based on sequence alone and most importantly designs specific mutations to decrease polyreactivity. We demonstrate successful use of our software on three polyreactive nanobodies, including AT118i4h32, a nanobody antagonist of the angiotensin II type I receptor (AT1R)^37^, where we reduce polyreactivity without compromising binding affinity or target-specific pharmacology. This sequence-based approach is a generally useful tool for prioritizing nanobody clones identified in selection experiments and the approach is in principle fully applicable to large libraries of conventional antibodies as well.

## Results

### Enrichment of naïve library for polyreactive clones

**Fig. 1:**
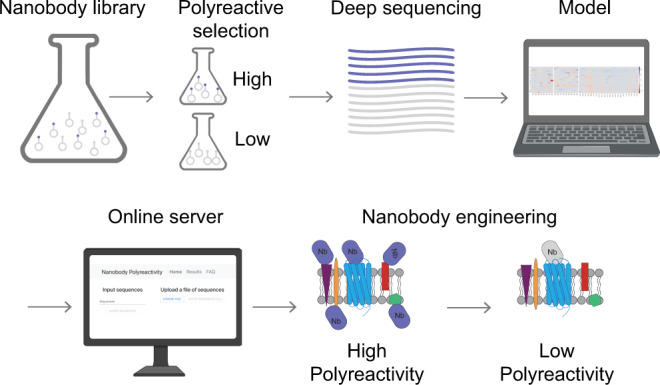
Development of a computational tool to assess and mitigate polyreactivity. Starting from a large, naïve synthetic nanobody library, pools of nanobodies with low and high polyreactivity were isolated. Machine learning models were trained on deep sequencing data from these pools to learn sequence features of low and high polyreactive nanobodies. These algorithms were incorporated into software that quantitatively predicts polyreactivity levels and recommends substitutions that reduce it. Created with BioRender.com.

Unlike previous analyses of antibody polyreactivity that primarily focused on clinical candidates^30–32^, clones enriched for antigen binding^24^, or primarily focused on the contribution of V_H_ CDR3 to antibody polyreactivity^25,28^, we designed experiments to assess polyreactivity of clones from a naïve synthetic yeast display library. The yeast display library contains >2 × 10^9^ unique nanobody clones that mimic a naïve llama immune repertoire in CDR sequence composition and CDR3 length and possesses moderate diversity in the CDR1 and CDR2 regions and extensive diversity in the CDR3 region^7,33^. We identified polyreactive clones that bound to detergent-solubilized *Spodoptera frugiperda* (Sf9) insect cell membranes (Fig. 1)^21^. This mixed protein polyspecificity reagent (PSR) is compatible with sorting large pools of antigen naïve clones to determine global contributions to polyreactivity in an unbiased manner^26^ and is well validated against other measures of polyreactivity for conventional antibodies^2,21,22^. Notably, PSR binding correlates with poor pharmacokinetics, a liability that is often discovered in late-stage clinical development and attributed to high polyreactivity^2^, and is commonly employed to detect polyreactivity^23,38–41^. We used Magnetic-Activated Cell Sorting (MACS) to enrich for polyreactive clones and deplete non-expressing clones from the library. Following MACS, distinct populations of clones with high and low polyreactivity were isolated by Fluorescence-Activated Cell Sorting (FACS) (Supplementary Fig. 1a, b).

**Fig. 2:**
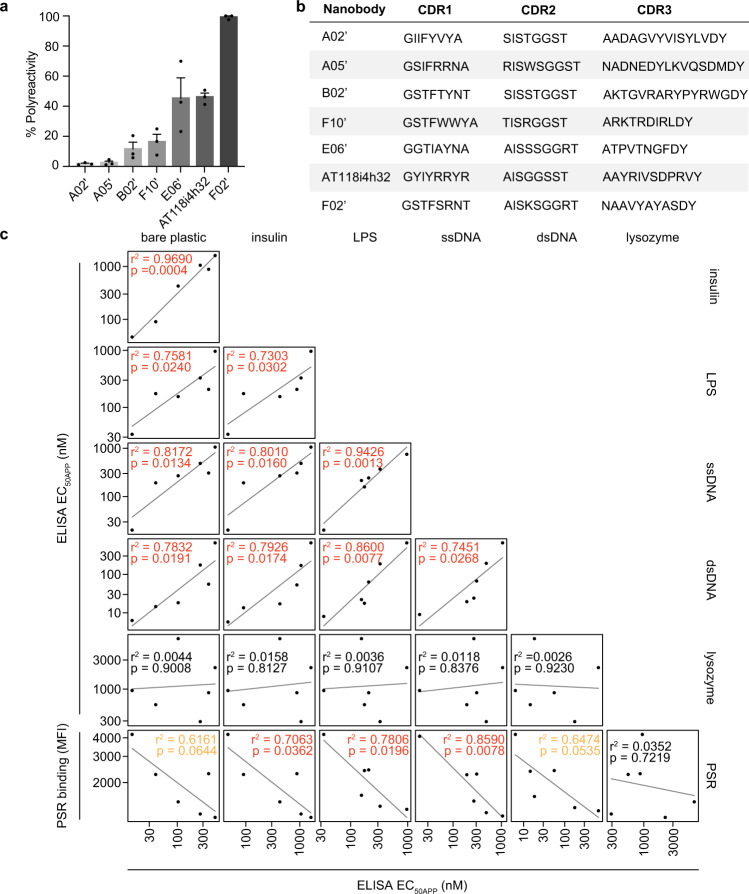
Properties of purified nanobodies exhibiting varying degrees of polyreactivity. **a** *Spodoptera frugiperda* (Sf9) insect cell PSR staining of single nanobodies isolated from FACS sorts. Data are mean +/− SEM of three independent biological experiments performed in technical triplicate. Polyreactivity levels are normalized with respect to the highest clone (Nb F02’). **b** CDR sequences of isolated nanobodies. **c** Direct ELISA assays measured the apparent EC_50_ (EC_50APP_) of five index set members and nanobody AT118i4h32 to the specified reagents. Non-specific binding, indicated by low EC_50APP_ values, correlates with strong binding to PSR. ELISA data are representative of two independent experiments, each performed in technical triplicates.

As PSR staining is typically used to analyze polyreactivity of conventional antibodies rather than nanobodies, we validated PSR performance on nanobodies. To ensure that we had not simply selected for nanobodies that bound to highly abundant molecules in insect cell PSR, we stained our low and high polyreactivity FACS pools with PSR derived from human embryonic kidney (HEK293) cells and observed comparable levels of staining for each clone (Supplementary Fig. 1c–f). We then recombinantly expressed six nanobodies with varying levels of polyreactivity from our FACS sorted pools and assessed polyreactivity by conventional direct ELISA assays against lysozyme, double-stranded DNA (dsDNA), single-stranded DNA (ssDNA), insulin, lipopolysaccharide (LPS), and bare plastic (Fig. 2, Supplementary Fig. 2a–f), which are commonly employed to assess antibody polyreactivity^15,16^. ELISA polyreactivity assays performed using different reagents correlated well with one another (r^2^ values between 0.745 and 0.969, *p* < 0.05) with the exception of lysozyme (r^2^ values between 0.003 and 0.016, *p*-values between 0.8127 and 0.9230), which did not correlate with the other reagents. Furthermore, direct ELISA assays strongly correlated with insect cell PSR (r^2^ values between 0.616 and 0.859) except for lysozyme which exhibited a very weak correlation (r^2^ = 0.035). The correlations between insulin, LPS, and ssDNA direct ELISA assays to insect cell PSR staining were highly significant (*p* < 0.05), while bare plastic and dsDNA direct ELISA assays were modestly significant (*p* < 0.10). Lysozyme direct ELISA assays did not significantly correlate with insect cell PSR staining (*p* = 0.7219). Overall, the ELISA experiments support that the pools of nanobodies selected by PSR staining possess high and low levels of polyreactivity. We then employed Affinity-Capture Self-Interaction Nanoparticle Spectroscopy (AC-SINS) as a third orthogonal technique to validate PSR performance at assessing nanobody polyreactivity (Supplementary Fig. 2g). AC-SINS measures the tendency of antibodies to self-associate and has previously been correlated to other polyreactivity measures for antibodies^22^. We observe that three nanobodies with low insect cell PSR staining do not self-associate in AC-SINS assays, while four nanobodies that stain strongly with insect cell PSR exhibit self-association and wavelength shifts greater than 5 nm, a previously reported cutoff for antibody self-association causing developability issues^42^. Given this validation, we deep-sequenced the two FACS sorted pools and obtained 65,147 unique low polyreactivity sequences and 69,155 unique highly polyreactive sequences that contained 51,308 and 59,623 distinct CDR regions.

### Development of computational method

**Fig. 3:**
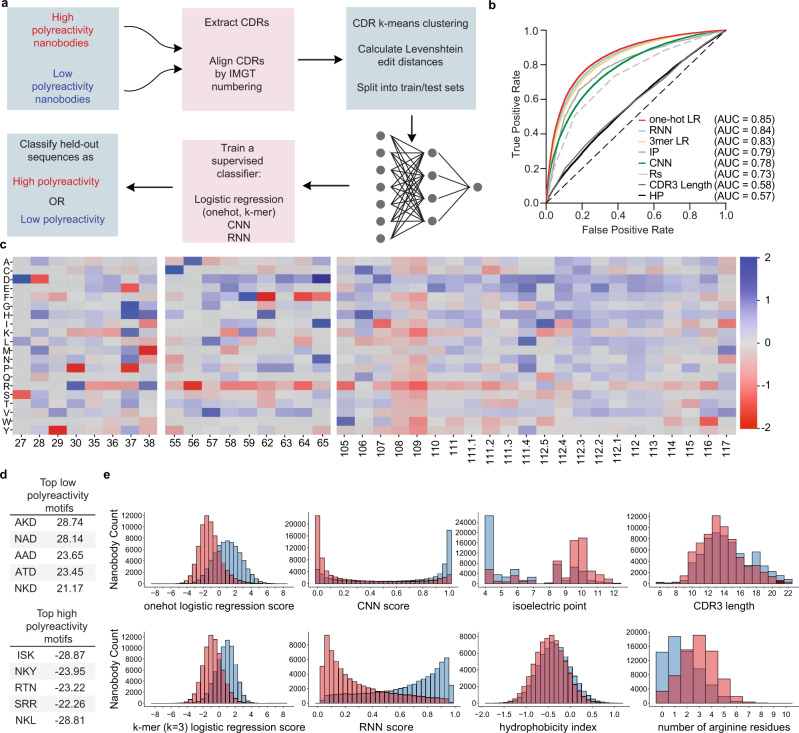
Development of computational models to predict polyreactivity. Supervised models were trained on pools of high and low polyreactivity sequences. **a** Pipeline of computational model development from raw NGS data to held-out predictions with sequence clustering for rigorous validation. **b** Comparison of supervised models (one-hot and k-mer logistic regression, RNN, CNN) and biochemical properties such as hydrophobicity, isoelectric point, CDR3 lengths, and number of arginine residues. **c** Trained parameters of a one-hot logistic regression model, showing which amino acids at specific positions are most predictive of high polyreactivity and low polyreactivity (red, negative score and blue, positive score, respectively). **d** Polyreactivity scores of top motifs learned from a k-mer logistic regression model that are most predictive of low and high polyreactivity (top and bottom, respectively). **e** Separation of high and low polyreactivity nanobodies by each of the models and biochemical properties displayed in panel b.

We developed computational models trained on the sequences from the FACS-sorted pools to classify nanobodies as possessing high or low polyreactivity. We constructed a suite of supervised, discriminative models that can separate high and low polyreactivity sequences (Fig. 3a, b). These models include a logistic regression model of a one-hot embedding of the CDR sequences, a logistic regression model of a k-mer embedding (*k* = 3) of the CDR sequences, a convolutional neural network (CNN), and a recurrent neural network (RNN). The one-hot logistic regression model learns weights for each amino acid type at each position in the CDR sequences that are most predictive of polyreactivity; the k-mer logistic regression learns weights for each motif (lengths 1, 2, and 3) that are most predictive of polyreactivity, irrespective of where they occur within a given CDR sequence. Convolutional neural networks use convolutional filters to learn spatial information (e.g., an amino acid and its neighboring residues). Recurrent neural networks capture sequential information (e.g., the probability of a residue given the previous residues). For the one-hot logistic regression and for the CNN, we align the CDR sequences using the IMGT numbering scheme with ANARCI^43^. The k-mer logistic regression and the RNN methods do not require aligned CDR sequences. In order to test the generalizability of our models, we clustered the nanobody sequences using k-means clustering to generate five clusters of sequences, which we used to build train and test splits. These splits and careful selection allowed us to avoid overly-optimistic prediction accuracies that result from the test sets overlapping with or being similar to the training sets^44^. Specifically, we ensured that all sequences in the test sets were more than 10 edit distance (Levenshtein distance) and possessed only ~75% sequence similarity in the CDR sequences from each other (Fig. 3a).

The one-hot logistic regression, k-mer logistic regression, and RNN models performed well at classifying distant nanobody sequences as high or low polyreactivity, achieving 0.85, 0.83, and 0.84 Area Under Curve (AUC) respectively (Fig. 3b). Whereas, the CNN (AUC = 0.78, Fig. 3b) achieved similar performance to metrics as described previously in literature, such as isoelectric point^23,29–31^ and the number of arginine residues^25,27,28,32^ (AUCs of 0.79 and 0.73, respectively, Fig. 3b). Consistent with previous literature^22,30^, we found that hydrophobicity, as described by the hydrophobicity index^45^, is not strongly predictive of polyreactivity (AUC = 0.57, Fig. 3b). However, CDR3 length, which is a reported feature of polyreactive antibodies^23,30^ is not highly predictive of nanobody polyreactivity (AUC of 0.58, Fig. 3b). Score and measurement distributions of the nanobody sequences for each of these metrics, separated by labeled class are displayed in Fig. 3e.

In addition to the models’ robust performance, sequence features learned by the logistic regression methods are easily interpretable. A distinct advantage of the one-hot logistic regression model is its ability to produce a picture of amino acid contribution to polyreactivity at each position of nanobody CDR sequences (Fig. 3c). In agreement with previous findings, we find that acidic residues in CDRs 2 and 3 are characteristic of low polyreactivity clones. The presence of arginine residues across all CDRs, and lysine, tryptophan, or tyrosine in CDR3 specifically contribute to higher polyreactivity. Despite the overall enrichment of arginine and tryptophan in polyreactive clones, the position-specific analysis provided by the one-hot model indicates that low polyreactivity clones tolerate arginine in positions 30 and 38 of CDR1 and tryptophan in position 105 in CDR3. Furthermore, we observe that acidic residues strongly contribute to polyreactivity at CDR1 positions 28 and 37, in contrast to their general polyreactivity-lowering tendency. We also find that certain positions can accommodate many amino acids without impacting polyreactivity (i.e., CDR2 position 58 and CDR3 position 106) while other positions only tolerate a narrow subset of amino acids (i.e., CDR3 108 and 109). These results inform future nanobody library design by suggesting which positions should be fixed or broadly diversified.

The k-mer logistic regression model provides additional insight into sequence dependencies on the local level in high or low polyreactivity clones (Fig. 3d). K-mer motifs containing negatively charged residues such as glutamate and aspartate are highly associated with low polyreactivity sequences, and positively charged residues such as arginine and lysine are predicted to contribute to polyreactivity, in concordance with the predictions of the one-hot logistic regression model. These motifs differ from previously reported polyreactive motifs that were enriched in glycine and the hydrophobic amino acids valine and tryptophan^25^. However, these previously reported motifs were derived from a library where only CDR3 was diversified. We proceeded to use the one-hot and k-mer logistic regression models for further analysis based on their accuracy and interpretability.

### Quantitative scoring of nanobody polyreactivity

**Fig. 4:**
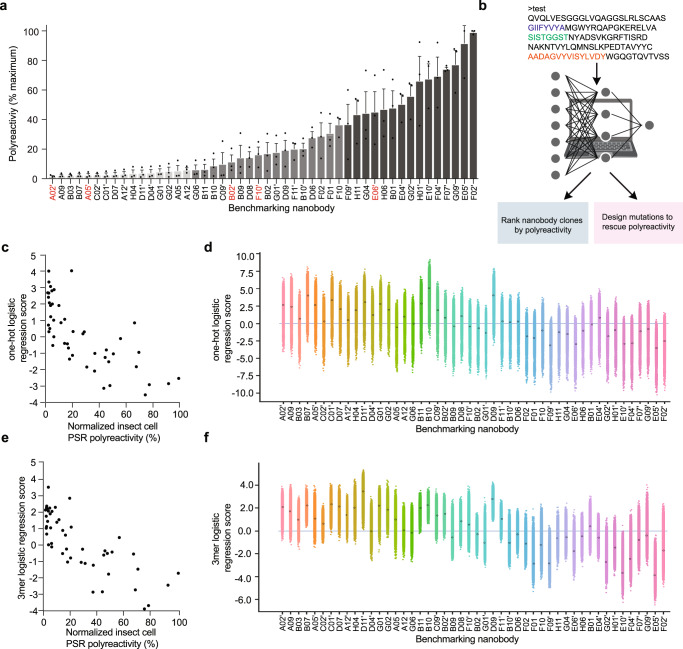
Validation of computational model for quantitative predictions of polyreactivity and generation of rescue mutations. **a** Generation of an index set of polyreactivity mutants by *Spodoptera frugiperda* (Sf9) insect cell membrane protein polyspecificity reagent **(**PSR) staining of yeast displaying 48 unique nanobodies isolated from MACS enrichment as well as non-reactive and polyreactive FACS pools. Data are mean +/− SEM of three independent biological experiments performed in technical triplicate. **b** New nanobody sequence(s) can be input into a webserver, which will output computational predictions of polyreactivity and biochemical properties of the sequence(s). It is also possible to input a nanobody sequence to retrieve top scoring rescue mutations predicted to decrease polyreactivity. **c**, **e** The one-hot logistic regression model and k-mer logistic regression model trained on the full NGS dataset from FACS sorts with PSR binding were used to test quantitative predictions and rankings of the index set of clones spanning a wide range of polyreactivity levels (as measured by PSR binding) (Spearman ρ of 0.77 and 0.79, respectively). A high score indicates low predicted polyreactivity, whereas a low score indicates increased polyreactivity. **d**, **f** An in silico double mutation scan (spanning substitutions, insertions, and deletions) was scored for predicted polyreactivity using both the one-hot logistic regression model and k-mer logistic regression model. From these in silico double mutation scans, a diverse set (spanning each CDR and combinations of CDRs) of high scoring mutations predicted to have low polyreactivity were selected as rescue mutations for experimental testing from two-parent clones, E10’ and D06. Created with BioRender.com.

In order to test if our model could go beyond predicting binary classification labels and quantitively score polyreactivity, we stained yeast expressing 48 nanobodies isolated from MACS and FACS pools with PSR to obtain an index set of sequenced clones with defined levels of polyreactivity (Fig. 4a, Supplementary Fig. 3a, Supplementary Table 1). Index set nanobodies were partitioned into three groups according to their level of polyreactivity: minimal polyreactivity (light gray), moderate polyreactivity (gray), and high polyreactivity (dark gray). Importantly, nanobody binding intensity to PSR reagent did not correlate with nanobody display level (r^2^ = 0.021, *p* = 0.391), suggesting that nanobody PSR binding intensities are directly comparable (Supplementary Fig. 3b). Furthermore, to confirm that the rank order was not skewed by PSR binding to unfolded nanobodies on the surface of yeast, the index set was stained with an anti-V_HH_ antibody, which recognizes the folded nanobody framework region (Supplementary Fig. 3c). Levels of anti-V_HH_ antibody staining are not correlated to insect cell PSR staining (r^2^ = 0.046, *p* = 0.1446, Supplementary Fig. 3d), indicating that unfolded clones do not confound our dataset. Finally, to verify that individual nanobody clones are not recognizing a specific component of the insect cell PSR reagent, we measured the polyreactivity of index set members using PSR reagent derived from solubilized HEK293 cell membranes. We found that insect cell and HEK293-derived PSR staining are highly correlated (r^2^ = 0.895, *p* < 0.0001), indicating that polyreactivity levels do not vary with PSR reagent type and therefore we are not strongly enriching for specific binders to one particular membrane component (Supplementary Fig. 3e).

Biophysical characteristics of clones in our index set were reflective of the learned features in our high and low polyreactivity pools. There is a modest but statistically significant correlation between PSR staining of the index set and nanobody isoelectric point (r^2^ = 0.390, *p* < 0.0001, Supplementary Fig. 3f). While nanobodies with low isoelectric points possess low polyreactivity, nanobodies with high pI values demonstrate a range of polyreactivity levels. In contrast, nanobody hydrophobicity index values are not correlated with polyreactivity (r^2^ = 0.036, *p* = 0.195, Supplementary Fig. 3g).

Of the 48 nanobodies, four were previously seen in our training set, so we did not include these in our quantitative tests. Each of the 44 remaining nanobodies had at least six mutations from any single nanobody sequence in the training set; the median of the minimum edit distance (a proxy for the number of mutations) of each of these index set nanobodies to the training set was 10 edit distance (the maximum similarity to the training set was 75% sequence identity). The correlation between the quantitative model predictions and the experimental binding scores to PSR are strong - about 85% of the maximum theoretical correlation (Spearman ρ of 0.77 and 0.79, for the one-hot and k-mer logistic regression models, respectively) (Fig. 4c, e). For comparison, the Spearman correlations between the three independent biological replicate experiments were 0.87, 0.87, and 0.95. Thus, our models trained on sequence pools of high and low polyreactivity nanobody CDR sequences are highly accurate for both classification and regression tasks for clones with distinct sequences.

### Model performance at predicting polyreactivity of closely related sequences

To determine if our computational models could accurately assess the influence of point mutations in single nanobody clones, we utilized the autonomous hypermutation yeast surface display error-prone DNA replication system (AHEAD)^46^ to rapidly evolve the four most polyreactive clones from our index set (Nb E05’, F02’, G09’, and F07’) to have reduced binding to the PSR reagent. Over the course of four AHEAD cycles involving nanobody hypermutation and FACS sorting, global PSR staining of the evolved nanobody population decreased (Supplementary Fig. 4). Deep sequencing analysis following the fourth FACS round revealed variation in the CDR regions of each of the four nanobodies.

A large proportion of the clones enriched by AHEAD are predicted to have reduced polyreactivity by both the one-hot and 3-mer logistic regression models. For the four clones, 97%, 67%, 69%, and 93% of the observed mutations are predicted to decrease polyreactivity by the one-hot logistic regression model, with similar decreases predicted by the k-mer logistic regression model (Supplementary Table 2). Furthermore, K31E^36^, A50T^55^, and R57P^64^ substitutions that arose in nanobody E05’ reflect the position-specific analysis provided by the one-hot logistic regression model, where K, R, and A are characteristic of polyreactive nanobodies at positions 36, 55, and 64 and all three substitutions are characteristic of clones with reduced polyreactivity (Fig. 3c). In a computational ranking of the polyreactivity of all 494 single amino acid substitutions using the one-hot logistic regression model in the CDR regions of E05’ found in our AHEAD experiment, from lowest to highest, R57P^64^ ranked 28^th^, K31E^36^ ranked 37^th^, and A50T^55^ was 101^st^. Overall, the AHEAD-based directed evolution experiment produced clones that our computational models predict to have reduced polyreactivity which suggests that our models can accurately score the polyreactivity of closely related sequences.

With confidence in our models’ performance on related clones, we employed our computational model to independently predict sequence substitutions to reduce polyreactivity of the highly polyreactive clone E10’ and moderately polyreactive clone D06 from our index set. We performed a comprehensive in silico single and double mutant scan, scored each sequence with both the one-hot logistic regression model and the k-mer logistic regression model (Fig. 4d, f), and ranked all the possible single and double mutants, including insertions and deletions, surrounding the seed sequence. We sampled the substitutions most likely to reduce polyreactivity (with the exception of a cysteine substitution which could disrupt disulfide bond formation) by selecting diverse mutations across residue types and positions that are contained within a single CDR and span each of the possible combinations of different CDR regions. Furthermore, if there was a mutation indicated to decrease polyreactivity by the k-mer logistic regression that scored similarly according to the one-hot logistic regression model, we selected the sequence with a higher k-mer logistic regression score to take into account local sequence dependencies. We selected the three top-scoring single mutations for each of the CDR regions, the top scoring double mutants within a single CDR region, and the top scoring double mutants spanning two CDR regions where at least one of the individual single mutations had not already been tested in a different combination (Supplementary Table 3).

**Fig. 5:**
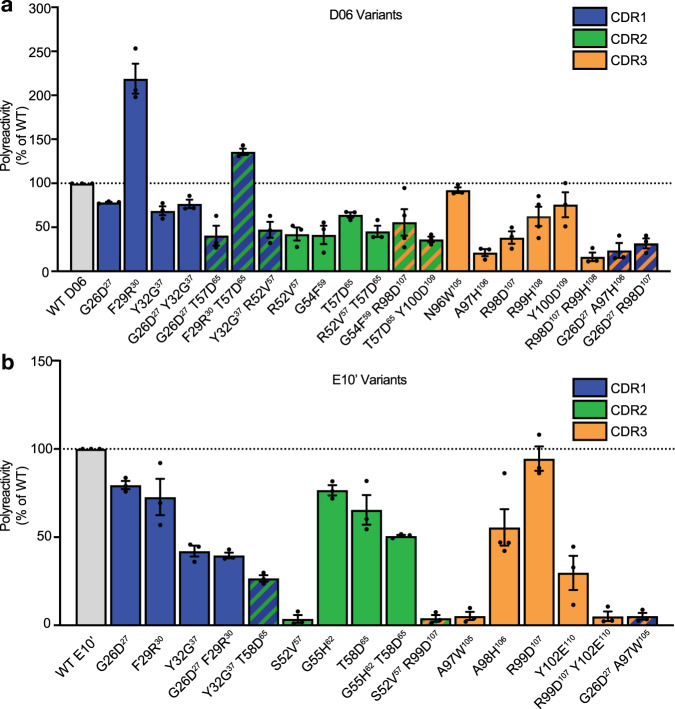
In silico designed substitutions reduce nanobody polyreactivity. **a** Polyspecificity reagent (PSR) staining of yeast displaying D06 variants. For the moderately polyreactive D06 nanobody, 18 out of 21 variants that were computationally designed to decrease polyreactivity reduced levels of binding to insect cell PSR staining. Data in **a** comprise the mean +/− SEM of at least three independent experiments, each performed in technical triplicate. **b** PSR staining of yeast displaying E10’ variants. For the highly polyreactive E10’ nanobody, 15 out of 16 computationally predicted single and double substitutions reduced binding to PSR reagent. Data in **b** comprise the mean +/− SEM of at least three independent experiments, each performed in technical triplicate. Substitutions to CDRs 1, 2, and 3 are colored in blue, green, and orange.

For the moderately polyreactive nanobody D06, 18 out of 21 variants that were computationally designed to decrease polyreactivity reduced levels of binding to insect cell PSR staining (Fig. 5a). More stringently, 11 out of 21 mutations exhibited at least two-fold reductions in polyreactivity. Although substitutions in each of the CDR regions were able to lower polyreactivity, CDR3 appeared to drive polyreactivity most strongly, as the largest reductions in polyreactivity occurred from variations in the CDR3 region including A97H^106^ and R98D^107^ R99H^108^.

For the highly polyreactive E10’ nanobody, 15 out of 16 computationally predicted single and double substitutions reduced binding to PSR reagent (Fig. 5b). 9 out of the 16 substitutions reduced polyreactivity by at least 50%, including mutations in each of the three CDR regions. Strikingly, the R99D^107^ Y102E^110^ clone, which was predicted to have the lowest polyreactivity value using the k-mer logistic regression model has very low polyreactivity by experimental PSR staining.

### Polyreactivity reduction of a functional clone

**Fig. 6:**
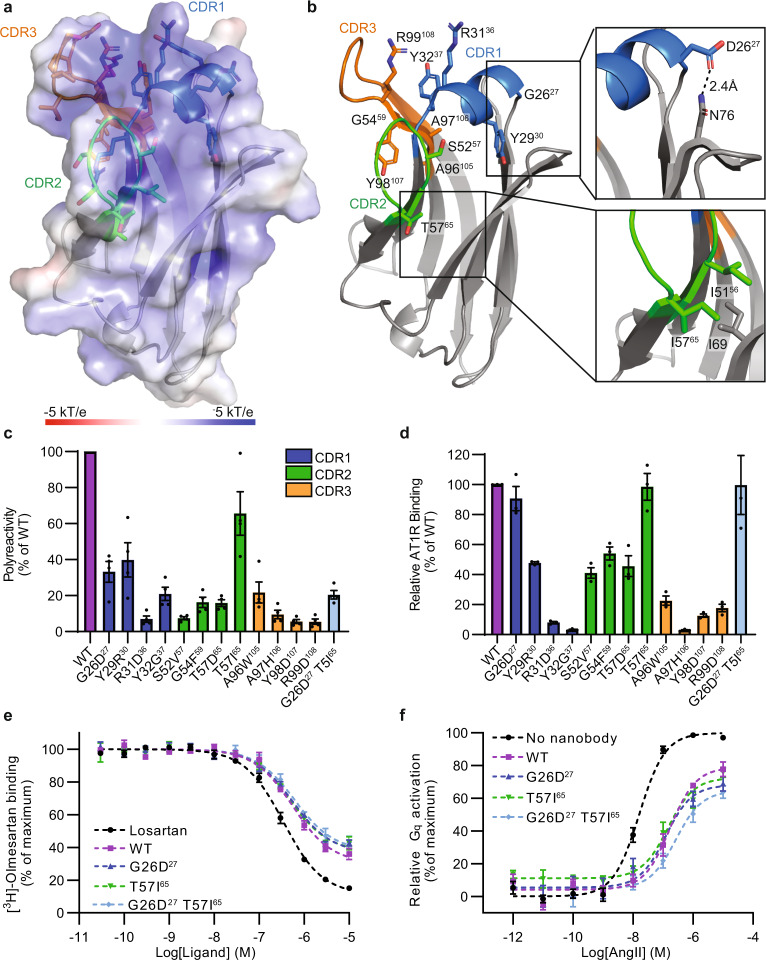
Development of AT118i4h32 variants with reduced polyspecificity. **a** Electrostatic surface of AT118i4h32. CDR1, CDR2, and CDR3 are colored blue, green, and orange. All positions substituted to produce variants of AT118i4h32 with reduced polyreactivity are shown in sticks with atomic coloring **b** AT118i4h32 structure as colored in a. G26D^27^ and T57I^65^ substitutions are boxed. **c** PSR staining of yeast displaying AT118i4h32 variants. All amino acid substitutions decrease polyreactivity. Data in c comprise the mean +/− SEM of four independent experiments, each performed in technical triplicate. CDRs are colored as in **a**. **d** Binding of AT118i4h32 variants to HEK293 suspension cells expressing FLAG-AT1R. Cells were stained with AT118i4h32-V5-His variants, AlexaFlour-488 conjugated anti-FLAG, and AlexaFlour-647 conjugated anti-V5 antibodies, then analyzed by flow cytometry. Data in d is the average of three independent experiments performed in technical triplicate, error bars are shown as SEM. **e** Radioligand competition binding of AT118i4h32 variants or the small molecule antagonist losartan and [^3^H]-olmesartan to AT1R in cell membranes. Like WT AT118i4h32, the G26D^27^, T57I^65^, and G26D^27^ T57I^65^ variants compete with olmesartan for binding to the AT1R. Data in e is the average of three independent experiments performed in technical triplicate, error bars are shown as SEM. **f** Suppression of Gq-mediated inositol monophosphate production by AT118i4h32 in response to AngII stimulation. HEK293 suspension cells expressing FLAG-AT1R were treated with 5 μM AT118i4h32 or no nanobody prior to AngII stimulation. Data in d is the average of three independent experiments performed in technical triplicate, error bars are shown as SEM. K_i_ values are reported in Supplementary Table 6.

We next tested if our model could be employed to decrease the polyreactivity of a nanobody clone that was independently selected for antigen specificity. AT118i4h32 is a nanobody antagonist for the angiotensin II type 1 receptor (AT1R), a G protein-coupled receptor (GPCR) that is a central regulator of blood pressure and renal function. Importantly, AT118i4h32 was humanized through the incorporation of eleven amino acid substitutions to AT118 to make it resemble human V_H_3-23 and thus AT118i4h32 possesses a distinct framework compared to nanobodies in the synthetic library. AT118i4h32 directly competes with the binding of small molecule and peptide ligands to the AT1R and is active in vivo, reducing mouse blood pressure to a comparable degree as the clinically used angiotensin receptor blocker losartan^37^. Although pharmacologically intriguing, AT118i4h32 is highly polyreactive in the PSR assay and has a high pI value (9.6), which is characteristic of polyreactive antibodies. Furthermore, a crystal structure of AT118i4h32 displays large patches of positive charge on the protein surface (Fig. 6a, Supplementary Table 4) and enrichment of both solvent exposed arginine and hydrophobic residues in the CDR regions (Fig. 6a).

We analyzed the sequence of AT118i4h32 and selected twelve single amino acid substitutions scattered throughout each CDR predicted to reduce polyreactivity based on the one-hot logistic regression model (Supplementary Table 3). AT118i4h32 variants were displayed on the surface of yeast and all showed reduced levels of PSR binding (Fig. 6c). Neutralizing the highly basic patch composed of R30^35^, R31^36^, and R99^108^ on the surface of AT118i4h32 (Fig. 6a) with R31D^36^ and R99D^108^ substitutions substantially reduces AT118i4h32 polyreactivity. Notably, introduction of an additional arginine residue with the Y29R^30^ substitution, which introduces a RRR sequence motif into CDR1, reduces polyreactivity, further demonstrating that arginine’s contribution to polyreactivity is position-dependent, which is captured through our machine learning models.

To assess the effects of these substitutions on antigen binding, AT118i4h32 variants were recombinantly expressed in *E. coli* and purified to evaluate AT1R binding by flow cytometry (Fig. 6d). Two AT118i4h32 variants, G26D^27^ and T57I^65^, retained at least 80% of wild-type binding levels to the AT1R. Combination of the G26D^27^ and T57I^65^ substitutions retained high levels of binding to the AT1R and yielded a clone with a modest decrease in PSR binding compared to the G26D^27^ variant (Fig. 6c), bringing the overall level of polyreactivity close to that of the clinically approved nanobody drug Cablivi/caplacizumab^47^ (Supplementary Fig. 5a). Additionally, the G26D^27^ T57I^65^ variant has reduced binding to the panel of bioreagents compared to the wild-type nanobody in ELISA assays (Supplementary Fig. 5b–g). AT118i4h32 variants containing G26D^27^ and T57I^65^ maintain affinity for AT1R (Supplementary Fig. 5h, i) and the ability to act as receptor antagonists, displacing small molecule orthosteric antagonists (Fig. 6e) and suppressing receptor signaling upon angiotensin II (AngII) stimulation (Fig. 6f).

To investigate how the G26D^27^ T57I^65^ substitutions alter AT118i4h32’s structure and contribute to reduce polyreactivity, we crystallized AT118i4h32 G26D^27^ T57I^65^ and solved the structure at 1.6 Å resolution (Fig. 6b, Supplementary Table 4). The T57I^65^ substitution is located at the end of CDR2. I57^65^ forms more favorable hydrophobic interactions with neighboring I51^56^ and I65 side chains than T57^65^. In the case of AT118i4h32, maintaining this hydrophobic interaction is essential for antigen recognition, as the T57D^65^ substitution diminished AT1R binding two-fold (Fig. 6d). While the T57I^65^ mildly decreases polyreactivity, AT118i4h32 variants containing the T57I^65^ substitutions had slightly decreased thermal stability (Supplementary Table 5), indicating that reduced polyreactivity is not necessarily correlated with thermal stability.

Residue D26^27^, found at the N-terminus of helical CDR1, forms a hydrogen bond with the side chain of framework residue N76 in all eight copies of the nanobody in the crystal structure’s asymmetric unit (Fig. 6b). This hydrogen bond rigidifies the CDR1 position and may reduce the flexibility of the nanobody’s CDR regions. Additionally, the G26D^27^ substitution improves AT118i4h32’s stability; we observed a five-fold increase in AT118i4h32 G26D^27^ yield from *E. coli* and a two-degree increase in melting temperature of the G26D^27^ variant (Supplementary Table 5) over wild-type levels. Corresponding G26D^27^ substitutions reduced the polyreactivity of nanobodies D06 and E10’. Despite occurring in just 0.05% of sequences from the naïve repertoire of seven llamas^48^ (1.12 million unique nanobody sequences), the G26D^27^ substitution may be both beneficial and tolerated in many sequence contexts and may broadly reduce polyreactivity by reducing the conformational flexibility of the CDR regions^49^.

### Expansion of computational methods using deeper sequencing

Upon examination of corresponding substituted positions in D06, E10’, and AT118i4h32 we observe some substitutions reduce polyreactivity in all clones, such as G26D^27^, whereas other mutations dramatically reduced polyreactivity of some nanobodies (i.e., E10’ A97W^105^ and AT118i4h32 A96W^105^) while having little to no effect in another clone (i.e., D06 N96W^105^). This suggests that position dependency is critical for polyreactivity, which may be more accurately captured with a larger data set. Therefore, we sought to improve our in silico method using deeper sequencing data. Through additional rounds of FACS selection, we collected 1,221,800 unique low polyreactivity clones and 1,058,842 unique high polyreactivity clones. We trained our suite of supervised classification models on this extended dataset and included analysis of an extra position at the end of CDR2, which has some variability in the synthetic nanobody library, but was not included in the initial analysis.

To test classification accuracy, we clustered the sequences into 10 clusters using a k-means algorithm for train/test splits, and again limited our training dataset to sequences with at least 10 mutations as compared to any sequence in the test sets. We achieved comparable classification AUCs to the logistic regression and RNN models trained on the original FACS sorts (one-hot logistic regression: 0.83, 3-mer logistic regression: 0.83, RNN: 0.84) (Supplementary Fig. 6a). The convolutional neural network model received a significant performance boost (CNN: 0.83 compared to 0.78 AUC previously) (Supplementary Fig. 6a). For the larger dataset, we see that the models that capture more complexities in sequences, such as the CNN and RNN, have higher accuracies, suggesting that there are meaningful dependencies in nanobody sequences that contribute to polyreactivity beyond site-specific amino acid contributions and/or 3-mer motifs and would allow us to make more accurate predictions to reduce polyreactivity for individual sequences. Furthermore, for each of these models we see an improved correlation (Spearman ρ) of polyreactivity scores with the index set measurements (one-hot logistic regression: 0.87, 3-mer logistic regression: 0.86, CNN: 0.88, RNN: 0.88) (Supplementary Fig. 6b–e). The majority of substitutions applied to clones D06, E10’, and AT118i4h32 are still predicted to decrease polyreactivity across the four models trained on the deeper FACS sequencing experiments (37, 37, 41, and 23 out of 45 mutations for one-hot logistic regression, k-mer logistic regression, CNN, and RNN respectively; for the RNN in particular, most mutations that were not predicted to decrease polyreactivity had very small changes in predicted signal, Supplementary Table 3).

As a resource to the field, we provide open-access use of our polyreactivity prediction software on our webpage (http://18.224.60.30:3000/). The webserver allows users to input a nanobody sequence(s) in FASTA format and outputs the aligned nanobody sequence with IMGT numbering using ANARCI^43^, along with biochemical properties of the sequence, including isoelectric point, hydrophobicity, CDR definitions (IMGT), CDR lengths, and computational predictions of polyreactivity scores using the one-hot logistic regression models that were trained for the design of rescue mutations. Polyreactivity values predicted by the server are displayed in the context of our index set (Fig. 4) and can be compared with clinical candidates (Supplementary Table 7) to assess developability.

## Discussion

Previous work has identified some biophysical characteristics of polyreactivity, but these studies have generally been performed on relatively small sets of antibody sequences and focused on improving a single antibody scaffold rather than providing a generalizable method to mitigate polyreactivity. Here, we designed and conducted high-throughput experiments to capture diverse clones that were not influenced by other selection pressures, facilitating an unbiased analysis of nanobody polyreactivity. Starting with a large naïve synthetic library mimicking the llama immunological repertoire, we isolated large pools of high and low-polyreactivity nanobody clones based upon binding to the mixed-protein PSR reagent. Our models are over 80% accurate in discriminating between clones with high and low polyreactivity (Fig. 3b), rank levels of polyreactivity with high fidelity (Fig. 4), and reliably identify amino acid substitutions that reduce polyreactivity (Figs. 5 and 6c).

Since our models were built upon experiments that were intentionally designed to interrogate sequence contributions to polyreactivity, they are highly accurate at measuring polyreactivity. In accordance with previous studies, our results show that arginine generally promotes nanobody polyreactivity while glutamate and aspartate usually decrease polyreactivity. We also report unexpected mutations that decrease polyreactivity including AT118i4h32 A96W^105^ and A97H^106^. We find that amino acid contributions to polyreactivity are highly position-dependent and more nuanced than broad generalizations suggest. This finding is in agreement with a recent independent study that analyzed polyreactivity of a subset of antibodies^24^. Furthermore, our computational models’ ability to accurately quantify polyreactivity from sequence identity allows detection and reduction of polyreactivity of existing clones. More complex models including the CNN and RNN models also allowed us to evaluate dependencies of amino acids in different locations in nanobodies to polyreactivity. We find such dependencies contribute to polyreactivity, indicating that both local and global characteristics of nanobodies influence their degree of polyreactivity.

We provide to the community an easy-to-use webserver that encapsulates our computational methods. These methods can guide antibody discovery campaigns at many points in the discovery pipeline and are especially useful for evaluating polyreactivity in fully synthetic antibody selections, which lack in vivo filtering for polyreactivity. For instance, our software predicted amino acid substitutions to reduce polyreactivity of the single clone AT118i4h32. In this case, the clone rescue approach allowed us to retain strict pharmacological function for this intriguing clone, which can be difficult to maintain during experimental selection rounds. Moreover, the polyreactivity of a list of antigen binders can be prospectively ranked to efficiently prioritize clones from large pools of NGS sequencing data during selection campaigns. Our method is especially powerful in instances where prior structural information describing the nanobody antigen interaction is available. We found that substitutions in each of the CDR regions of D06, E10’, and AT118i4h32 reduce polyreactivity, suggesting that each CDR region contributes to polyreactivity. Therefore, if a certain CDR region is critical for antigen recognition, substitutions in alternative CDR regions can potentially compensate in reducing polyreactivity. In addition, our success in reducing polyreactivity of AT118i4h32, where the humanized framework region differs from clones in the training set, indicates that our methods are applicable to nanobodies from a range of sources and can be fully integrated with existing computational tools to reduce immunogenicity^50^.

As recently reported^24,51,52^, similar approaches could be applied to fully characterize sequence features of polyreactive conventional antibody clones. These methods can be expanded by analyzing large antigen-naïve libraries and adding in the three light-chain CDRs and germline gene choice as additional factors for polyreactivity prediction and optimization. Learned sequence features can be applied to future library design to create next-generation synthetic antibody and antibody-fragment libraries containing clones with reduced polyreactivity. Overall, these models, derived from a large sequence space, can be combined with affinity data, gathered during the selection process, to computationally predict antibodies with high antigen specificity and low polyreactivity, without additional experimental effort.

## Methods

### Generation of insect cell membrane polyreactivity reagent

Insect cell membrane polyreactivity reagent was generated as described previously^21^. Briefly, 250 mL of Sf9 insect cells at a density of 4 × 10^6^ cells/mL were pelleted, washed in 100 mL PBS + 1% BSA followed by 30 mL Buffer B (50 mM Hepes pH 7.2, 150 mM NaCl, 2 mM CaCl_2_, 5 mM KCl, 5 mM MgCl_2_, 10% glycerol). The cell pellet was resuspended in 3x pellet volume of Buffer B with a protease inhibitor tablet (Roche) and lysed with a dounce homogenizer. The membrane fraction was pelleted by centrifugation at 40,000 x *g* for 1 hour, washed with 1 mL Buffer B, and resuspended in 3 mL of Buffer B with dounce homogenization. Total protein was quantified using the DC protein assay (Biorad) following manufacturer’s instructions. The membrane fraction was diluted to ~1 mg/mL in Buffer B and biotinylated with 200 μM NHS-LC-Biotin for three hours at 4 °C. 20 mM Tris pH 8 was added to quench excess NHS-LC-Biotin. The biotinylated membrane fraction was centrifuged at 40,000 x *g* for 1 hour and the pellet was washed 5 times with Buffer B and resuspended in 3 mL Buffer B + 10% glycerol by dounce homogenization, and total protein was quantified by the DC protein assay (Biorad). The membrane fraction was diluted to 1 mg/mL in solubilization buffer (50 mM Hepes pH 7.2, 150 mM NaCl, 2 mM CaCl_2_, 5 mM KCl, 5 mM MgCl_2_, 10% glycerol, 1% DDM, 1 x protease inhibitor pH 7.2) and stirred overnight at 4 °C. The mixture was centrifuged for 40,000 x *g* for 1 hour. Total protein in the supernatant containing the solubilized membrane fraction was quantified using the DC protein assay and aliquots were flash-frozen and stored at −80 °C.

### Yeast sorting

A yeast surface display library containing >2 × 10^9^ synthetic nanobody sequences was used where each amino acid position is diversified based on the natural llama immunological repertoire^33^. Nanobodies are tethered to the yeast cell surface on a synthetic stalk^7^ from a vector encoding nourseothricin resistance^33^ in *Saccharomyces cerevisiae* BJ5465. Nanobody expression was induced for 36–48 hours in dropout medium without tryptophan (-Trp) supplemented with galactose. 5 × 10^9^ yeast cells were stained with 10% PSR in selection buffer (20 mM Hepes pH 7.5, 100 mM NaCl, 0.1% DDM, 0.01% CHS 0.05% BSA, 5 mM CaCl_2_, 10 mM maltose) at 4 °C for 1 hour. Cells were spun down, resuspended in 4.5 mL selection buffer, and incubated with 500 µL streptavidin conjugated microbeads (Miltenyi) for 20 min at 4 °C. Cells were washed with 5 mL selection buffer and applied to a LS column (Miltenyi). The column was washed with 8 mL selection buffer. 2.8 × 10^7^ yeast clones were collected in the MACS elution and subjected to a round of FACS. 5 × 10^7^ yeast cells were stained with 10% PSR in selection buffer for 1 hour at 4 °C, washed with selection buffer, and stained with a 1:100 dilution of Alexaflour-647 conjugated anti-HA antibody to detect nanobody expression and Alexaflour-488 conjugated streptavidin (Biolegend) to detect biotinylated PSR positive cells for 15 min. Cells were washed with selection buffer and resuspended in selection buffer for FACS on a SONY SH800 cell sorter. Gates to detect low and high polyreactivity clones were set based upon cells stained with Alexaflour-647 conjugated anti-HA antibody and Alexaflour-488 conjugated streptavidin. For initial experiments 5 × 10^7^ total yeast cells were sorted. 3.7 × 10^6^ low polyreactivity clones and the most polyreactive clones (top ~1% containing 3.2 × 10^6^ clones) were collected. To obtain additional sequencing data the MACS enriched library was subjected to additional rounds of cell sorting to collect 4.6 × 10^7^ highly polyreactive clones and 9.8 × 10^5^ low polyreactivity clones. Cells from the MACS elution and low and high poyreactivity FACS sorted populations were plated on -Trp media to obtain single clones. Flow cytometry gating figures were generated in FlowJo (10.8.1).

### Deep sequencing

The nanobody sequences were amplified from the low and high polyreactivity populations via colony PCR. Media was aspirated from 4 × 10^6^ pelleted yeast cells. Cells were microwaved for 1 min on high power twice. Cells were resuspended in 1x Q5 High-Fidelity master mix containing 0.3 mM forward (GTTCAATTGGACAAGAGAGAAGCT) and reverse primers (GTAATCTGGAACATCGTATGGGTA). Cells were subjected to a 4 min incubation at 95 °C and DNA was amplified following the manufacturer’s protocol. Amplified DNA was gel extracted and evaluated via Illumina MiSeq in a 2 × 250 paired-end sequencing reaction.

### NGS analysis and sequence processing

Fastq sequences from deep sequencing were processed using the FastQC, Trimmomatic, FASTX-Toolkit programs. Sequences were translated to protein sequences using the Biopython package and only nanobody sequences were retrieved by selecting for the highly conserved final beta strand sequence. The nanobody sequences were aligned using ANARCI with standard IMGT numbering to identify the CDR regions. For our dataset of sequences to train the supervised models, we limited nanobody sequences to sequences with a CDR1 length of 8, a CDR2 length of 8 or 9 (9 or 10 in the deeper sequencing exploration, when we include an additional position at the end of CDR2 to include more variability), and CDR3 lengths between 6 and 22. These processing steps leave us with 65,147 unique low polyreactivity sequences and 69,155 unique highly polyreactive sequences that contained 51,308 and 59,623 distinct CDR regions.

### Supervised model development

The CDR regions were used to build four different types of supervised models: a one-hot logistic regression model, a k-mer logistic regression model, a CNN, and an RNN. The logistic regression models were built using the scikit-learn python package. For the one-hot logistic regression model and CNN model, the sequences were processed into aligned one-hot encoding vectors of amino acids per position (via IMGT numbering). For the RNN, sequences were processed into non-aligned one-hot encoding vectors (padded at the ends of sequences to the longest length). For the k-mer logistic regression model, sequences were processed into vectors of k-mers ranging from single amino acids (1-mer) to 3-mer motifs. The CNN and RNN models were written in pytorch. The CNN has three convolutional layers (first layer: 1D-convolution (channel dimension size 20 → 32) with kernel size of 3, BatchNorm, and ReLU; second layer: 1D-convolution (channel dimension size 32 → 64) with kernel size of 3, BatchNorm, ReLU, and MaxPool with kernel size of 3, stride of 3; 1D-convolution (channel dimension size 64 → 128) with kernel size of 3, BatchNorm, ReLU, and MaxPool with kernel size of 3, stride of 1) followed by a fully connected layer and a final sigmoid for binary classification and was trained using the Adam optimizer. The RNN has two layers and a hidden size of 128. For splitting sequences by clusters and sequence identity sci-kit learn KMeans clustering and python-levenshtein package was used.

### Yeast plasmid transformation

pYDS plasmids were transformed into yeast following standard protocol^53^. 2x YPAD media was inoculated with *Saccharomyces cerevisiae* BJ5465 and grown to a density of 2 × 10^7^ cells/mL. Cells were harvested by centrifugation and washed with water five times. 1E^6^ cells were suspended in a 360 uL transfection mix containing 33.3% PEG3350, 100 mM lithium acetate, 0.28 mg/mL salmon sperm carrier DNA, and 1 μg of the pYDS plasmid encoding the nanobody, synthetic stalk, and nourseothricin resistance cassette. The transformation mixture was incubated at 42 °C for 40 min. Yeast cells were isolated by centrifugation, washed with water, and resuspended in YPAD. After a 1-2 hour outgrowth at 30 °C without shaking, cells were plated on YPAD supplemented with 100 μg/mL nourseothricin.

### Anti-V_HH_ Antibody Staining

Polyreactivity index panel yeast were grown in -Trp + Glu media for two days at 30 °C and induced in -Trp + Gal media at 25 °C for two days. After induction, 1 × 10^6^ yeast cells were washed with DDM selection buffer and were stained with a 1:100 dilution of Alexafluor-488 conjugated Monorab Rabbit Anti-Camelid V_HH_ Antibody (Genescript) and 1:100 dilution of Alexafluor-647 conjugated anti-HA antibody. Following an additional wash, analytical staining was performed using a BD Accuri C6 flow cytometer.

### Recombinant nanobody expression and purification

Recombinant nanobodies containing a C-terminal V5 epitope and hexahistidine tag were cloned into pET26b and amino acid substitutions were introduced using the QuikChange lighting site-directed mutagenesis kit (Agilent). Plasmids were transformed into *E. coli* BL21(DE3) in Terrific Broth (RPI) supplemented with 4% glycerol and 50 ug/mL kanamycin to an OD600 of 1–2 at 37 °C and cooled to 17–20 °C for one hour. Protein expression was induced using 0.2 mM isopropyl β-D-1-thiogalactopyranoside (IPTG, Gold Biotechnology) overnight. Bacterial pellets were resuspended in room temperature SET lysis buffer (200 mM Tris pH 8, 500 mM sucrose, 500 μM EDTA) with gentle stirring for 20 min, followed by addition of 2x volume ice cold DI H_2_O, 5 mM magnesium chloride, and 1 μL benzonase nuclease (Sigma-Aldrich) for 1 hour. Cellular debris was removed by centrifugation at 14,000 x g for 30 minutes. Following centrifugation, 100 mM sodium chloride was added to the supernatant with stirring for 15 min and the supernatant was filtered using glass microfiber filters (Fisher Scientific). Clarified lysate was passed over Protein A resin (Gold Biotechnology) equilibrated with Protein A wash buffer (10 mM sodium phosphate pH 7.5, 100 mM sodium chloride). Then, the column was washed with 10 column volumes Protein A wash buffer, and nanobody was eluted using 10 columns Protein A elution buffer (100 mM sodium phosphate pH 2.5, 100 mM sodium chloride) directly into 1 column volume 2 M Hepes pH 8. The Protein A column eluate was passed over a Ni-NTA (Qiagen) column equilibrated with Ni-column wash buffer (20 mM Hepes pH 7.5, 150 mM sodium chloride). The column was then washed with 10 column volumes Ni-NTA wash buffer and eluted using 10 column volumes Ni-NTA elution buffer (20 mM Hepes pH 7.4, 150 mM sodium chloride, 200–400 mM imidazole). The eluate was then dialyzed overnight against SEC buffer (20 mM Hepes pH 7.5, 150 mM sodium chloride, 10% glycerol) and concentrated. Index set nanobodies were purified by size exclusion chromatography using a Superdex S-75 10/300 GL column (GE Healthcare) gel filtration system. Protein purity was assessed by SDS-PAGE.

### Nanobody polyreactivity ELISA assays

Direct ELISA assays were performed similarly to those reported previously. Briefly, high-binding Costar 96-well plates (Corning) were coated with 0.5 μg salmon sperm ssDNA (Abcam), calf thymus dsDNA (Sigma-Aldrich), lipopolysaccharide from *E. coli* (Sigma-Aldrich), chicken egg white lysozyme (Sigma-Aldrich), or 0.25 μg insulin (Fitzgerald) and incubated overnight at 4 °C. The next morning, plates were washed three times using wash buffer (PBS pH 7.5, 0.001% Tween), were blocked using blocking buffer (PBS pH 7.5, 0.1% Tween-20, 1 mM EDTA, 2% BSA) for two hours at room temperature, and then were washed three times with wash buffer. Following blocking, nanobodies (200 μL) were incubated at the indicated concentrations at room temperature in PBS pH 7.5 for two hours. After three more washes, plates were incubated with HRP-anti V5 antibody (Abcam ab1325, 1:10,000 dilution) in PBS + 2% BSA for one hour at room temperature. Plates then were washed three times with wash buffer and 1-Step ABTS substrate solution (100 μL, Thermo Scientific) was added to the plates, which were then incubated in the dark for 20 min. Stop solution (1% SDS in PBS, 100 μL) was added to each plate and absorbance at 405 nm was measured using a Spectromax M5 microplate reader. Results were analyzed in GraphPad Prism.

### AC-SINS experiments

AC-SINS experiments were performed as described previously^42^. Briefly, AffiniPure Goat Anti-Human IgG, Fcy fragment capture antibody (Jackson ImmunoResearch) and ChromoPure Goat IgG whole molecule non-capture antibody (Jackson ImmunoResearch) were dialyzed overnight into 20 mM sodium acetate pH = 4.3. Then, a 4:1 mass ratio of capture to non-capture antibody was prepared. A 9:1 ratio of gold nanoparticles to capture/non-capture antibody mixture was incubated overnight at room temperature and was treated with 0.1 μM PEG methyl ether thiol to block empty sites on the gold nanoparticles. Next, a 10x solution of gold nanoparticles was prepared by spinning gold nanoparticles down at 20,000 x g for 5 min and resuspending in a tenth of the original volume. For each sample tested, 5 μL of 10x gold nanoparticles and 45 μL of nanobody sample at 0.05 mg/mL were incubated together for 2 hours in the dark in a polypropylene plate. Absorbance was read using an Envision I spectrophotometer in 1 nm increments between 450 and 650 nm. A_max_ values were calculated by fitting experimental data with a 2nd order polynomial model and calculating the wavelength where the slope is equal to zero.

### AHEAD orthogonal replication

Nanobodies were amplified using primers PSR_Nb_F and PSR_Nb-R and cloned into the AHEAD integration plasmid (pAW240). The plasmids were linearized with ScaI and transformed into the AHEAD base strain as previously described^46^. At each cycle, 5 × 10^7^ cells were labeled with biotinylated insect cell membrane polyreactivity reagent and a HA epitope tag binding antibody as described above and subjected to FACS selection applying a gate that enriches for cells with reduced binding to PSR. The typical number of cells that were selected at each round was 400 out of 2 × 10^7^ sorted cells. The selected cells were sorted into 3 mL of SC – HLUW media and grown at 30 °C with 250 RPM shaking for 48 hours until saturation. Cells cultures were then induced for nanobody display by diluting them at a 1:20 ratio into SC -HLUW media containing 2% galactose instead of glucose and incubated at 20 °C for 48 hours. In preparation of next-generation sequencing, p1 plasmid was extracted, as previously described^54^, from yeast cultures after the FACS step of each AHEAD cycle. PCRs were performed with Q5 Master Mix (New England Biolabs Cat# M0492S) and primers NGS_p1_F and NGS_p1_R. Following PCR reactions, samples were PCR purified. Amplicon sequencing was performed by the Genewiz and the resulting sequences were analyzed using the methods described above.

### Polyspecifity Reagent Analytical Staining

Mutations in D06, E10’, and AT118i4h32 were introduced using the Quikchange Lightning mutagenesis kit (Agilent), and yeast were transformed using a standard transformation protocol. Polyreactive nanobody panel and mutant yeast were grown in -Trp + Glu media for two days at 30 °C and induced in -Trp + Gal media at 25 °C for two days. 1 × 10^6^ yeast were washed with DDM selection buffer, and were stained with a 1:10 dilution of either insect cell PSR reagent or Expi cell PSR reagent for 30 min at 4 °C with shaking. Following incubation with PSR reagent, yeast were washed with DDM selection buffer and were stained with a 1:100 dilution of Alexafluor-647 conjugated anti-HA antibody and 1:100 dilution of Alexafluor-488 conjugated streptavidin (Biolegend) for 15 min at 4 °C with shaking. Cells were washed once more with DDM selection buffer and analytical staining was performed using a BD Accuri C6 flow cytometer.

### AT118i4h32 AT1R binding assay

Expi293F cells stably expressing the tetracycline repressor^55^ were stably transfected with a wild-type human FLAG-AT1R containing plasmid (pCDNA Zeo-TetO) to create an inducible cell line, as previously described^56^. Expi293F TetR Zeo FLAG-AT1R cells were grown to 1.5–2 × 10^6^ cells/mL induced with 0.4 μg/mL doxycycline hyclate for 24 hours.

Cells were washed with cold flow assay buffer (20 mM Hepes pH 7.4, 150 mM NaCl, 0.1% BSA). 2.8 × 10^5^ cells were plated and stained with 20 nM of each AT118i4h32 variant with a C-terminal V5 epitope in flow assay buffer in 100 µL reaction volumes for 1 hour at 4 °C with gentle shaking. Cells were washed 2 times with flow assay buffer and subsequently stained with 100 nM of Alexaflour 488 conjugated M1-anti FLAG antibody and 1:200 of Alexaflour 647 conjugated anti-V5 antibody (ThermoFisher) in flow assay buffer + 1 mM CaCl_2_ for 20 minutes at 4 °C. Cells were washed once and resuspended in flow assay buffer + 1 mM CaCl_2_. Samples were analyzed on an BD Accuri C6 flow cytometer. Cells were gated for M1-positive singlets. Data were analyzed with BD Accuri C6 Plus software.

### AT118i4h32 saturation-binding experiments

For saturation-binding experiments cells were harvested, washed, and resuspended in flow assay buffer. 1 × 10^5^ cells were stained with varying concentrations of AT118i4h32 or AT118i4h32 G26D^27^ T57I^65^ containing a C-terminal V5 epitope tag for 1 hour at 30 rpm at 4 °C. Cells were then washed twice with flow assay buffer, supplemented with 1 mM CaCl_2_, and incubated with a 1:750 dilution of Alexa Fluor 647-labeled anti-V5 antibody (Invitrogen Thermo Fisher) and 100 nM Alexa Fluor 488-labeled M1 anti-FLAG for 20 min at 30 rpm at 4 °C. Cells were washed, resuspended in flow cytometry buffer with 1 mM CaCl_2_, and analyzed with a Cytoflex flow cytometer. AT1R expressing cells were gated for M1-positive singlets. Data were analyzed with BD Accuri C6 Plus software.

### AT1R signaling assay

Expi293F cells stably expressing a tetracycline inducible wild-type human FLAG-AT1R were diluted to 1.5–2 × 10^6^ cells/mL and induced with 0.4 μg/mL doxycycline hyclate for 24–28 hours. 2 × 10^4^ cells were plated into a low-volume 96-well plate, treated with 5 μM of each AT118i4h32 variant for 30 min at 37 °C, and stimulated with AngII for 1 hour at 37 °C. IP1 was detected with the IP-One Gq kit (CisBio) and read on a SpectraMax M5e plate reader (Molecular Devices).

### Radioligand binding assays

Cell membranes for radioligand binding experiments were prepared from Expi293F cells stably expressing tetracycline inducible wild-type human FLAG-AT1R. AT1R expression was induced at 2 × 10^6^ cells/mL with 0.4 μg/mL doxycycline hyclate for 30 hours. Cells were pelleted and washed with cold HBS. Cells were resuspended in 2.5 mL of 20 mM Tris pH 7.4 per gram of cell pellet with a protease inhibitor tablet and lysed by dounce homogenization (100x). Membranes were isolated by centrifugation at 50,000 x *g* for 20 min. Membranes were resuspended in 2.5 mL of 50 mM Tris pH 7.4, 12.5 mM MgCl_2_, 150 mM NaCl, 0.2% BSA + protease inhibitor table by dounce homogenization, flash frozen in liquid N_2_, and stored at −80 °C.

Membranes were incubated with nanobodies and 2 nM [^3^H]-olmesartan (American Radiolabeled Chemicals) in 50 mM Tris pH 7.4, 12.5 mM MgCl_2_, 150 mM NaCl, 0.2% BSA for 90 min at room temperature. Reactions were harvested on a GF/B filter soaked in water on a 96-well Brandel harvester and washed three times with cold water. Radioligand affinity was measured by saturation binding of [^3^H]-olmesartan in the presence and absence of 10 μM candesartan. Inhibitory constant (K_i_) values were determined through a one-site competition binding model in GraphPad Prism. Data represents the mean and SE of three independent biological replicates performed in triplicate.

### Protein crystallization and structure determination

AT118i4h32 with a N-terminal methionine and alanine and C-terminal His-tag was crystallized at 20 °C by sitting drop vapor diffusion from a 1:0.5 µL mixture of protein stock (10 mg/mL AT118i4h32 in 20 mM Hepes pH 7.4, 100 mM NaCl) and reservoir solution (16% PEG 4000, 10% isopropanol, 0.1 M sodium citrate pH 5.6). Crystals were flash-cooled directly from the drop in liquid N_2_.

Diffraction data were collected at 100 K on GM/CA beamline 23ID-D at the Advanced Photon Source (APS) at Argonne National Laboratory. Diffraction data were processed using XDS^57^. A camelid antibody 1YC7 was used to solve the structure of AT118i4h32 by molecular replacement using Phaser in the Phenix software suite^58^. The model was rebuilt using Autobuild and manually completed by iterative rounds of model building and refinement using Coot and Phenix.refine with 56 translation/liberation/screw groups. The structure was validated using Molprobity.

AT118i4h32 G26D^27^ T57I^65^ with a N-terminal methionine and alanine and C-terminal His-tag was crystallized at 20 °C by sitting drop vapor diffusion from a 0.5:1 µL mixture of protein stock (6.96 mg/mL AT118i4h32 G26D^27^, T57I^65^ in 20 mM Hepes pH 7.4, 150 mM NaCl) and reservoir solution (30% PEG 3350, 280 mM lithium citrate tribasic). Crystals were cryoprotected in 25% PEG 3350, 280 mM lithium citrate tribasic, 15% glycerol and then flash cooled in liquid nitrogen.

Diffraction data were collected at 100 K on GM/CA beamline 23ID-D at the Advanced Photon Source (APS) at Argonne National Laboratory. Diffraction data were processed using XDS^57^. The structure of AT118i4h32 was used to solve the AT118i4h32 G26D^27^ T57I^65^ crystal structure by molecular replacement using Phaser in the Phenix software suite^58^. The model was manually completed by iterative rounds of model building and refinement using Coot and Phenix.refine with 69 translation/liberation/screw groups. The structure was validated using Molprobity. Figures were prepared in PyMol^59^. All software was accessed through SBGrid^60^.

### Thermal shift assay

Differential scanning fluorimetry (DSF) experiments were carried out using a Quant Studio 6 real-time PCR machine (Applied Biosystems). 0.1 mg/mL of AT118i4h32 variants in HBS + 10% glycerol was mixed with Protein Thermal Shift Dye (Applied Biosystem) in a 1:100 (v/v) ratio of protein to dye. Samples were heated from 25–90 °C at a rate of 3 °C per minute. The fluorescence was detected with 470 +/−15 nm excitation and 586 +/−10 nm emission filters. All samples were measured by three biological replicates of technical triplicates. Fluorescence values were fit to the Boltzman equation and melting temperatures (Tm) were extracted from the inflection points of the curves in the Protein Thermal Shift Software (Applied Biosystems).

### Statistical methods

Prism software (GraphPad) was used to analyze data and perform error calculations. Data are expressed as arithmetic/geometric mean +/− SEM or arithmetic/geometric mean ± SD.

### Reporting summary

Further information on research design is available in the Nature Portfolio Reporting Summary linked to this article.

## Supplementary information

Supplementary Information

Reporting Summary

## Data Availability

The data that support this study are available from the corresponding author upon request. Coordinates and structure factors for the AT118i4h32 structures are deposited in the Protein Data Bank under accession codes 7T83 and 7T84. Source data are provided with this paper.

## Source data

Source Data

## Code availability

The code for scoring new sequences for polyreactivity, designing rescue mutations, training polyreactivity models, and calculating biochemical properties of a sequence can be found on github: https://github.com/debbiemarkslab/nanobody-polyreactivity, and the webserver is available here: (http://18.224.60.30:3000/).
