# Supplementary material for: An in silico method to assess antibody fragment polyreactivity
Source: Nat Commun. 2022 Dec 7;13:7554. doi: 10.1038/s41467-022-35276-4 (PMC9729196; doi:10.1038/s41467-022-35276-4)
Supplement: Supplementary file 1 — Supplementary Information [file 41467_2022_35276_MOESM1_ESM.pdf]

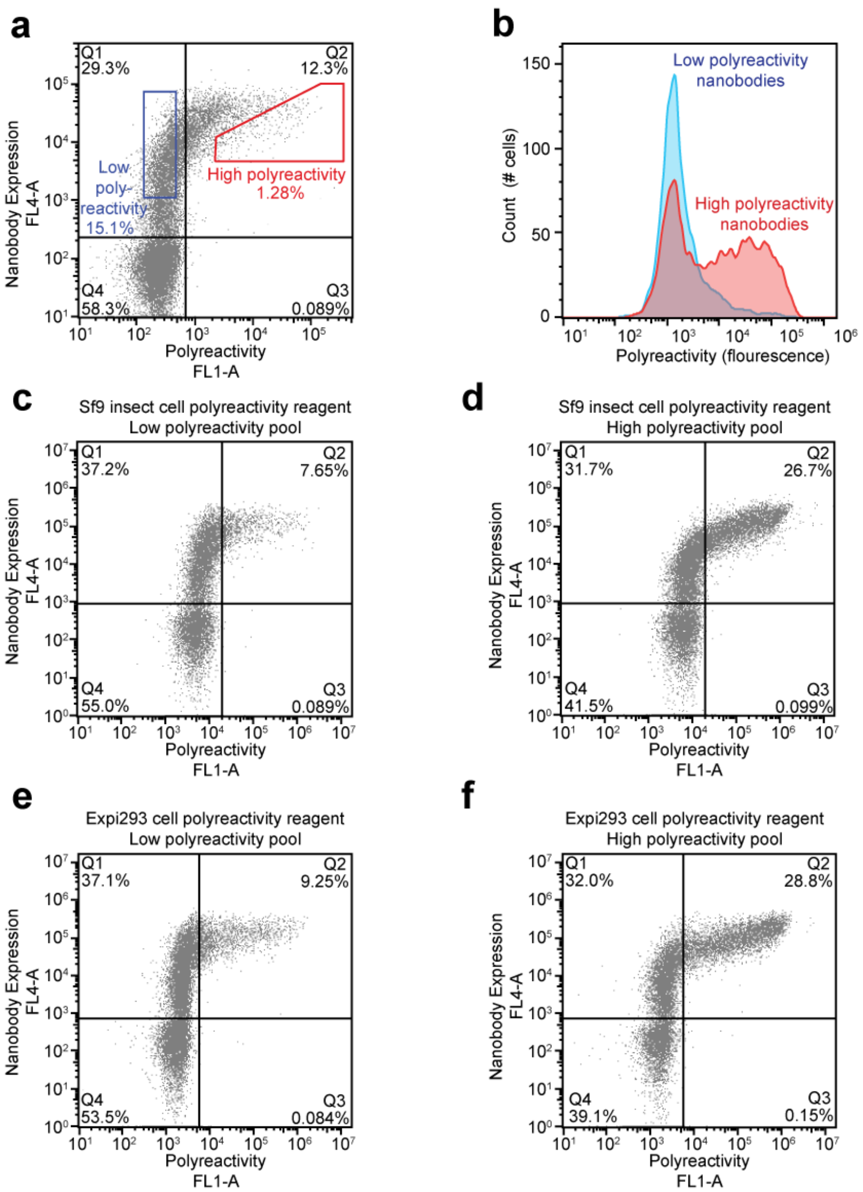

Supplementary Figure 1. FACS Gating strategies. a, the nanobody library enriched for PSR binders via MACS was stained with biotinylated insect cell PSR reagent, followed by AlexaFlour-647 conjugated αHA-antibody to assess nanobody expression on the yeast cell surface and AlexaFlour-488 conjugated streptavidin. Highly expressing yeast representing the low polyreactivity (PSR negative) and high polyreactivity (PSR positive) populations were collected. **b**, Analytical flow cytometry staining illustrates the difference in polyreactivity between the low polyrectivity and high polyreactivity nanobody pools. Low and high polyreactivity pools were concurrently stained with insect cell PSR reagent (**c-d)** and HEK293 PSR reagent (**e-f**), demonstrating a similar level of binding to both PSR reagents.

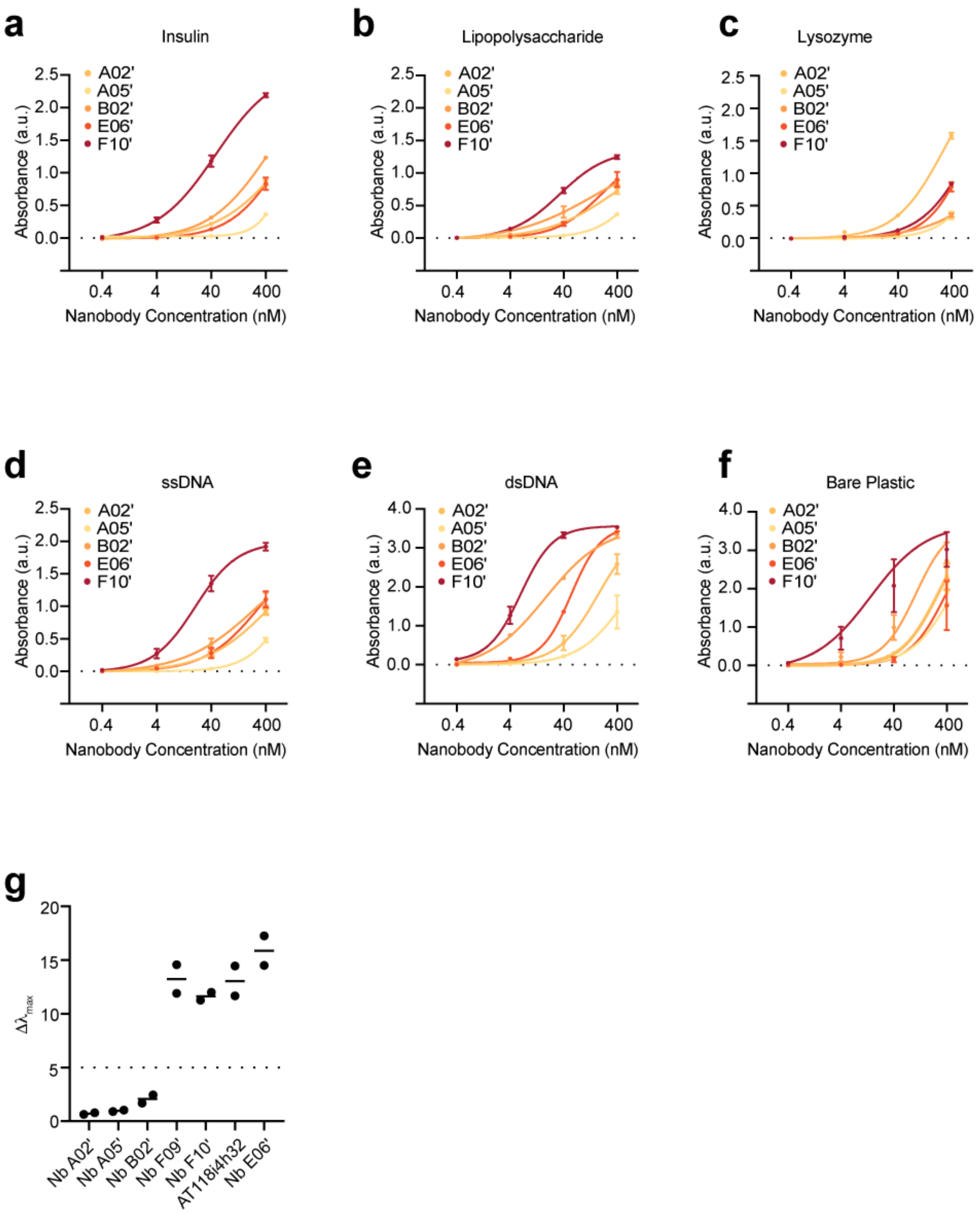

Supplementary Figure 2. ELISA assessment of polyreactivity. a-f, Polyreactivity of index panel members as measured by nanobody binding to the indicated reagents in direct ELISAs. Data are mean +/- SEM of two independent experiments, each performed with technical triplicates. **g,** Self association of index panel members as measured by AC-SINS. Data are the max wavelength shift observed and are representative of results obtained in two independent experiments.

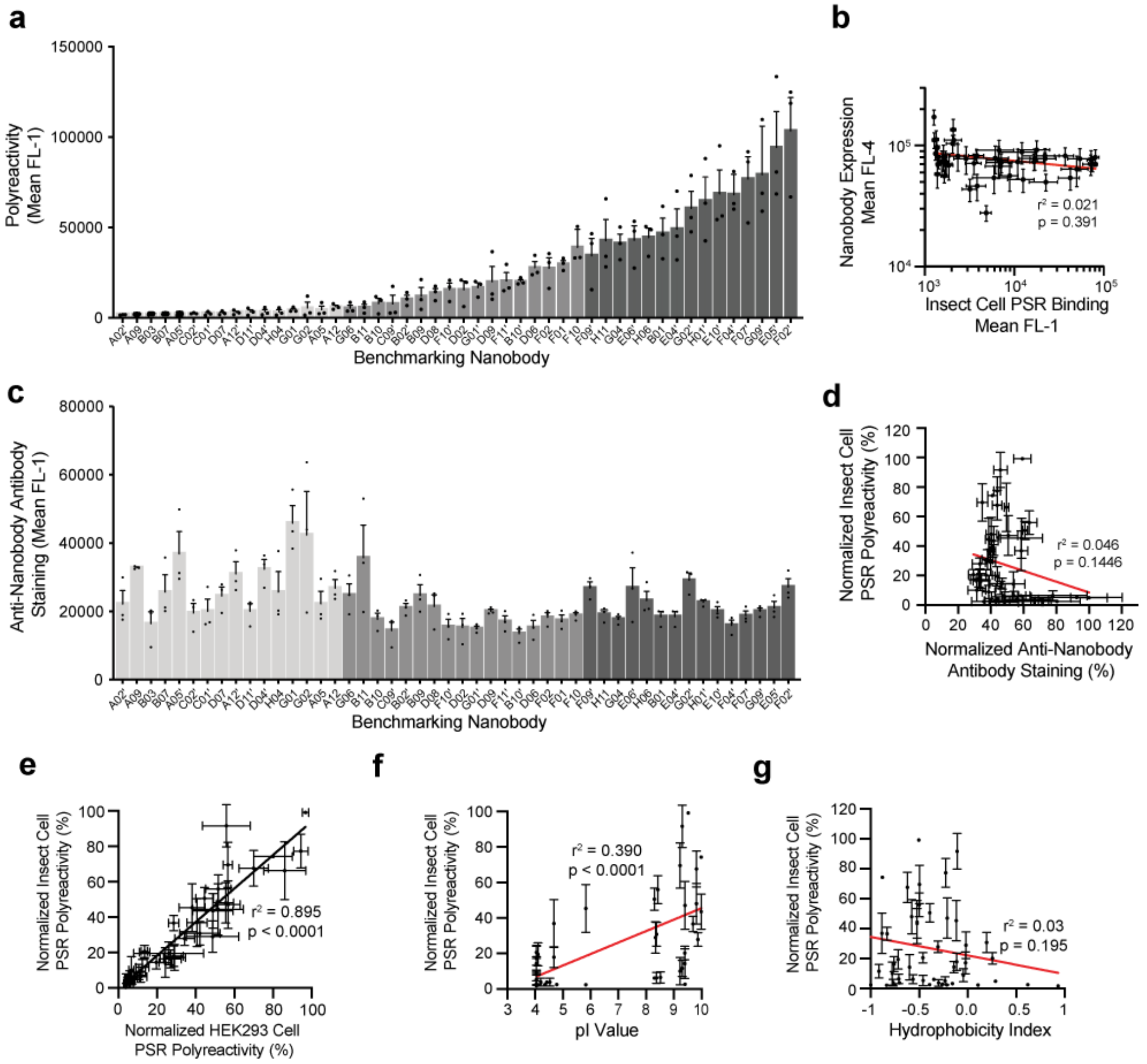

Supplementary Figure 3. Benchmarking nanobody set. a, Raw insect cell PSR staining of yeast displaying polyreactivity index panel members. Data are mean +/- SEM of three independent biological replicates performed in technical duplicate. **b,** Insect cell PSR staining is not correlated with nanobody expression. **c,** Anti-nanobody antibody staining of yeast displaying polyreactivity index panel members, indicating that all panel members are folded. Data are mean +/- SEM of three independent biological experiments performed in technical triplicate. **d,** anti-nanobody antibody staining is not correlated with insect cell PSR staining ($r^2 = 0.046$). **e,** Insect cell PSR staining of index panel members is well correlated with HEK293 cell PSR staining, suggesting that polyreactive panel members are not binding to specific proteins in insect cell membranes. HEK293 cell PSR staining data are mean +/- SEM of three independent biological experiments. **f,** Correlation between index panel nanobody pI values and insect cell PSR reagents. Nanobodies with low pI values tend to possess low polyreactivity, while nanobodies with high pI values exhibit a wider range of polyreactivity. **g,** Index panel nanobody hydrophobicity index values and insect cell PSR reagent staining are not correlated.

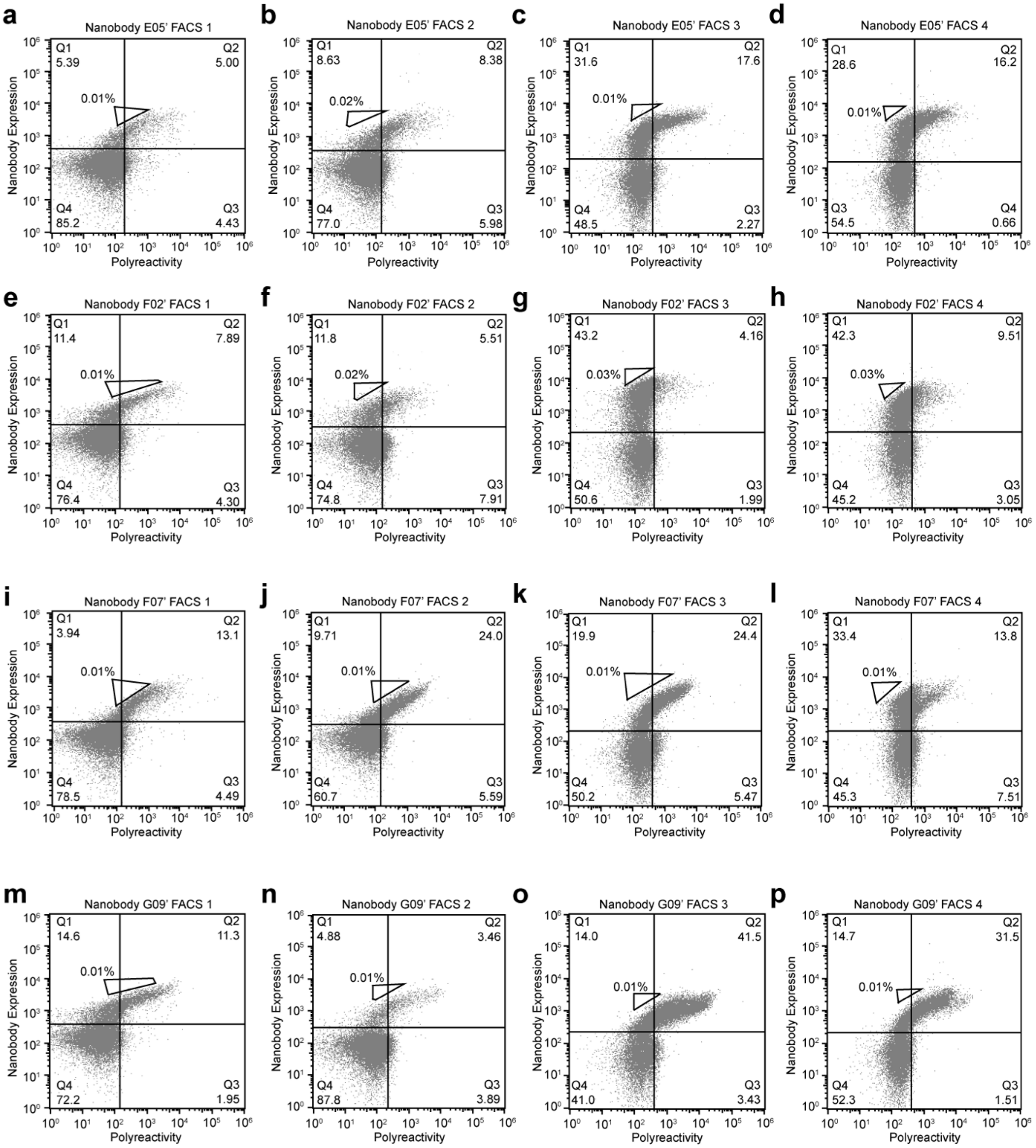

Supplementary Figure 4. Evolution of index panel nanobodies using the AHEAD system to reduce polyreactivity. a-d, Selected FACS plots showing polyreactivity reduction of nanobody E05’. **e-h,** Selected FACS plots showing polyreactivity reduction of nanobody F02’. **i-l,** Selected FACS plots showing polyreactivity reduction of nanobody F07’. **m-p,** Selected FACS plots showing polyreactivity reduction of nanobody G09’.

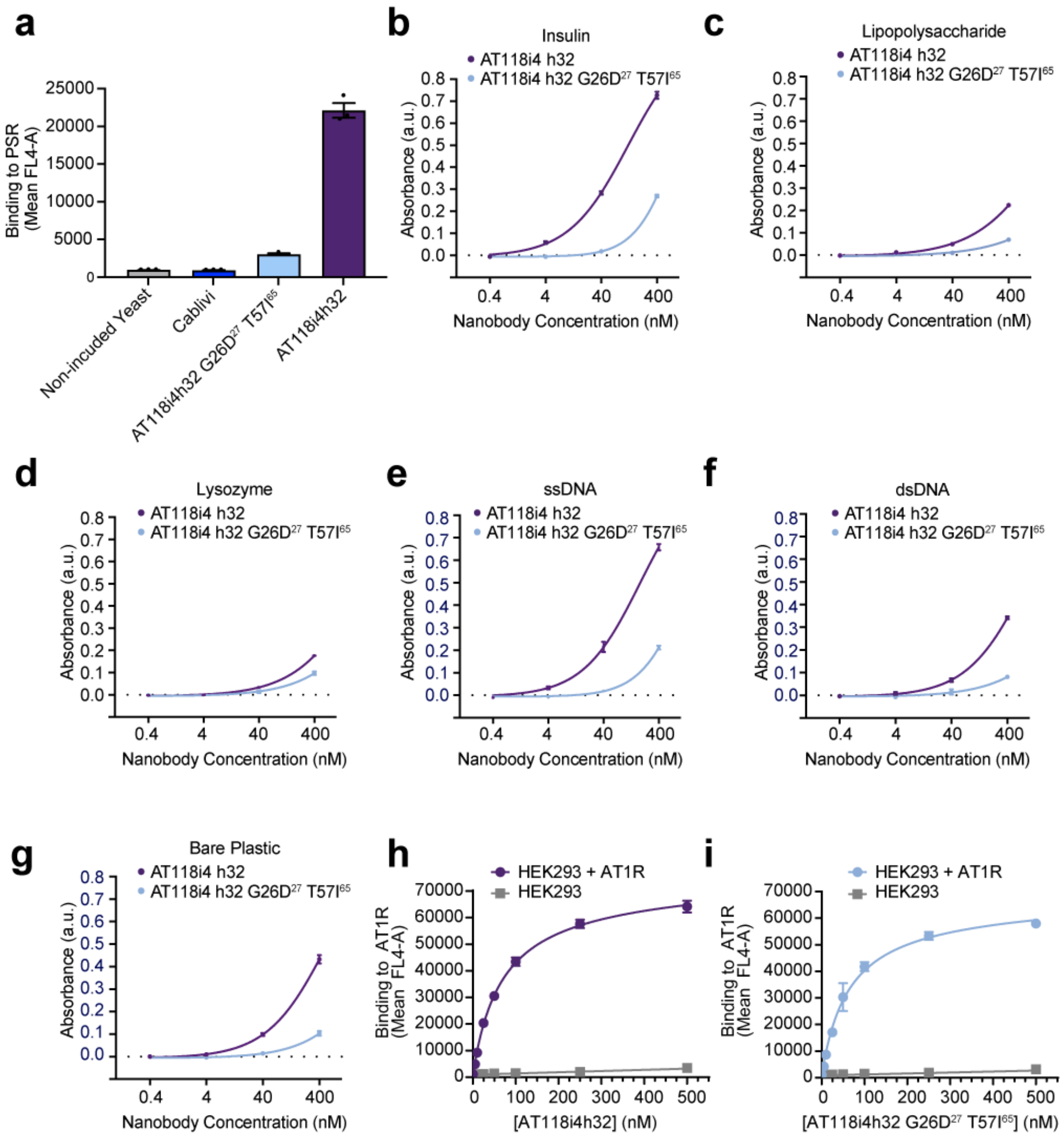

Supplementary Figure 5. Polyreactivity of AT118i4 variants. a, Polyreactivity of non-induced yeast not expressing nanobody, AT118i4h32, AT118i4h32 G26D^{27} T57I^{65}, and the clinically approved nanobody drug Cablivi, as measured by insect cell PSR staining. Data is representative of three independent experiments. Error bars represent SEM of three technical replicates**. b-g,** Polyreactivity of AT118i4 h32 and AT118i4 h32 G26D^{27} T57I^{65}, as measured by binding of AT118 variants to the specified reagents by direct ELISA assays. Data are mean +/- SEM and are representative of two independent experiments, each performed with technical triplicates. **h-i**, HEK suspension cells stably expressing AT1R were treated with AT118i4h32-V5-His (purple) or AT118i4h32 G26D^{27} T57I^{65}-V5-His (blue) and Alexa Flour 647-labeled anti-V5 antibody and analyzed by flow cytometry. Data are representative of three independent experiments. Error bars represent mean +/- SEM of three replicates. Non-specific binding was measured against wild-type HEK suspension cells. The measured K_d for AT118i4h32-V5-His is 58.3 +/- 6.1 nM and 54.0 +/- 7.4 nM for AT118i4h32 G26D^{27} T57I^{65}-V5-His.

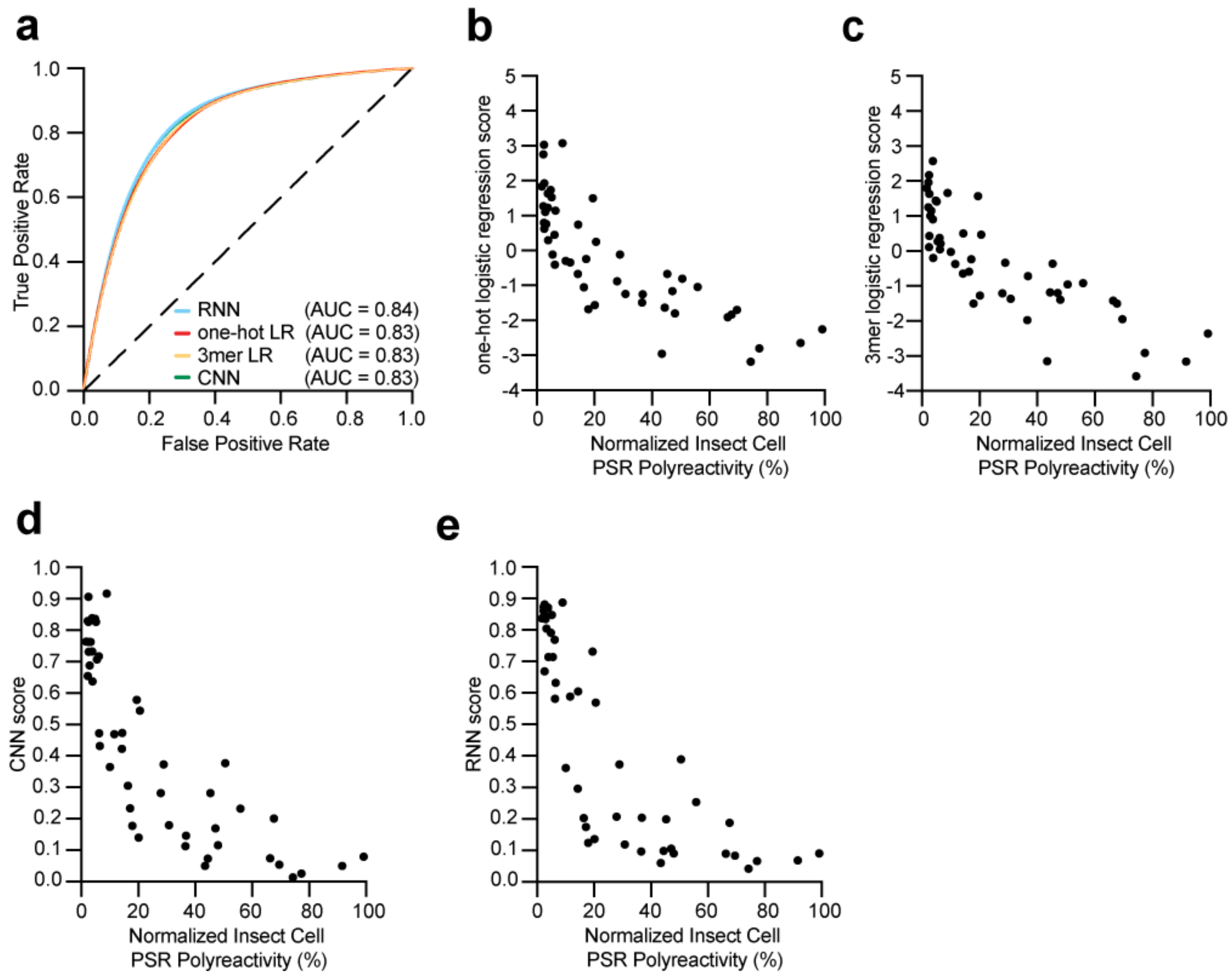

Supplementary Figure 6. Deep FACS training results. a, Comparison of supervised models (one-hot and k-mer logistic regression, RNN, CNN) trained on a deeper FACS sort of millions of high and low polyreactive sequences. Sequences were clustered into ten validation clusters and models were trained in cross-fold validation using only sequences greater than 10 mutations away from any test sequence in the training set. **b-e,** Quantitative predictions of polyreactive compared to the experimentally measured PSR levels of the index set of clones. The one-hot logistic regression, k-mer logistic regression, CNN, and RNN models were trained on the full NGS dataset (except for sequences that had the exact same CDR sequences as sequences in the index set) and were used to predict relative polyreactivity (spearman ρ_s of 0.87, 0.86, 0.88, 0.88) respectively.

Supplementary Table 1. Nanobody Sequences

**Name**	**Sequence**<br>(Bold = mutation from corresponding parent, only non-synonymous mutations are indicated; Italics = CDR3)
A02’	QVQLVESGGGLVQAGGSLRLSCAAS*GIIFYVYA*MGWYRQAPGKERELVA*SISTGGST*NYADSVKGRFTISRDNAKNTVYLQMNSLKPEDTAVYYC*AADAGVYVISYLVDY*WGQGTQVTVSS
C01’	QVQLVESGGGLVQAGGSLRLSCAAS*GSIFLLYA*MGWYRQAPGKERELVA*AITIGGST*NYADSVKGRFTISRDNAKNTVYLQMNSLKPEDTAVYYC*NAPEVIRPYFVAYDY*WGQGTQVTVSS
A05’	QVQLVESGGGLVQAGGSLRLSCAAS*GSIFRRNA*MGWYRQAPGKERELVA*RISWSGGST*YYADSVKGRFTISRDNAKNTVYLQMNSLKPEDTAVYYC*NADNEDYLKVQSDMDY*WGQGTQVTVSS
A09	QVQLVESGGGLVQAGGSLRLSCAAS*GKYNA*MGWYRQAPGKEREFVA*AISLGGSTT*YYADSVKGRFTISRDNAKNTVYLQMNSLKPEDTAVYYC*ARDGPYDAHSLEDDY*WGQGTQVTVSS
B07	QVQLVESGGGLVQAGGSLRLSCAAS*GFIFHYYV*MGWYRQAPGKERELVA*AITSDDS*TYADSVKGRFTISRDNAKNTVYLQMNSLKPEDTAVYYC*AVDVWYSTDHFSDYDY*WGQGTQVTVSS
H04	QVQLVESGGGLVQAGGSLRLSCAAS*GFIFRYNA*MGWYRQAPGKERELVA*TITSSDGST*YYADSVKGRFTISRDNAKNTVYLQMNSLKPEDTAVYYC*AEFWVGVYNHPGYDY*WGQGTQVTVSS
D07	QVQLVESGGGLVQAGGSLRLSCAAS*GSTLYGYV*MGWYRQAPGKERELVA*AITDSGGST*YYADSVKGRFTISRDNAKNTVYLQMNSLKPEDTAVYYC*AADYPYFYYVKSYDY*WGQGTQVTVSS
B03	QVQLVESGGGLVQAGGSLRLSCAAS*GRIFSGNA*MGWYRQAPGKERELVA*AITFSGGST*NYADSVKGRFTISRDNAKNTVYLQMNSLKPEDTAVYYC*NVASSDGYYYFAIGYGY*WGQGTQVTVSS
D04’	QVQLVESGGGLVQAGGSLRLSCAAS*GSTSWRNA*MGWYRQAPGKERELVA*AISTGGNT*YYADSVKGRFTISRDNAKNTVYLQMNSLKPEDTAVYYC*AAYDASQYGYDY*WGQGTQVTVSS
A12’	QVQLVESGGGLVQAGGSLRLSCAAS*GSTFYYYA*MGWYRQAPGKERELVA*VISWSGGS*TYADSVKGRFTISRDNAKNTVYLQMNSLKPEDTAVYYC*AAGAYKPVDTSDY*WGQGTQVTVSS
C02’	QVQLVESGGGLVQAGGSLRLSCAAS*GRTFTYNA*MGWYRQAPGKERELVA*RISFSTGST*YYADSVKGRFTISRDNAKNTVYLQMNSLKPEDTAVYYC*AVSTTSFGAQTGYGPYPYGY*WGQGTQVTVSS
D11’	QVQLVESGGGLVQAGGSLRLSCAAS*GSIFGDNA*MGWYRQAPGKERELVA*TITFRGAGT*YYADSVKGRFTISRDNAKNTVYLQMNSLKPEDTAVYYC*AKDYWSAWQNNGYDY*WGQGTQVTVSS
A05	QVQLVESGGGLVQAGGSLRLSCAGYAMGWYRQAPGKERELVA*TISGSGGST*YYADSVKGRFTISRDNAKNTVYLQMNSLKPEDTAVYYC*AAAYWDYSYEYYY*WGQGTQVTVSS
C09’	QVQLVESGGGLVQAGGSLRLSCAAS*GRIFGRNA*MGWYRQAPGKERELVA*AISWSGGN*TYYADSVKGRFTISRDNAKNTVYLQMNSLKPEDTAVYYC*AKDVSYPYKVTWHYQYDY*WGQGTQVTVSS

G01	QVQLVESGGGLVQAGGSLRLSCAAS*GIIFNGYA*MGWYRQAPGKERELVA*AITDDGTS*TYYADSVKGRFTISRDNAKNTVYLQMNSLKPEDTAVYYC*AADGGLVDFYY*WGQGTQVTVSS
G06	QVQLVESGGGLVQAGGSLRLSCAAS*GSTFGANT*MGWYRQAPGKERELVA*AISWSGGT*TYYADSVKGRFTISRDNAKNTVYLQMNSLKPEDTAVYYC*ARYKYPADYGY*WGQGTQVTVSS
B11	QVQLVESGGGLVQAGGSLRLSCAAS*GSTFSSNA*MGWYRQAPGKERELVA*SINSGDST*YYADSVKGRFTISRDNAKNTVYLQMNSLKPEDTAVYYC*AVVRFGTRPSHIRHTHEYYY*WGQGTQVTVSS
B10	QVQLVESGGGLVQAGGSLRLSCAAS*GSIFVYNA*MGWYRQAPGKERELVA*AITYSGDDT*YYADSVKGRFTISRDNAKNTVYLQMNSLKPEDTAVYYC*AAEYVEGVLSIYGRSWVYNTYDY*WGQGTQVTVSS
A12	QVQLVESGGGLVQAGGSLRLSCAAS*GRIFSGYA*MGWYRQAPGKERELVA*TITYTGGST*YYADSVKGRFTISRDNAKNTVYLQMNSLKPEDTAVYYC*NTSPYYVADLRYYY*WGQGTQVTVSS
G02	QVQLVESGGGLVQAGGSLRLSCAAS*GSILLPYA*MGWYRQAPGKERELVA*TISSSGGST*YYADSVKGRFTISRDNAKNTVYLQMNSLKPEDTAVYYC*AVEQYSNYLENDY*WGQGTQVTVSS
B09	QVQLVESGGGLVQAGGSLRLSCAAS*GSIFVDNA*MGWYRQAPGKERELVA*SITWRGGRT*SYADSVKGRFTISRDNAKNTVYLQMNSLKPEDTAVYYC*NKVSYRSWYYPAFDY*WGQGTQVTVSS
B02’	QVQLVESGGGLVQAGGSLRLSCAASG*STFTYNT*MGWYRQAPGKERELVA*SISSTGGST*YYADSVKGRFTISRDNAKNTVYLQMNSLKPEDTAVYYC*AKTGVRARYPYRWGDYDY*WGQGTQVTVSS
B02	QVQLVESGGGLVQAGGSLRLSCAAS*GSTFGLYA*MGWYRQAPGKERELVA*AITWSGGTT*YYADSVKGRFTISRDNAKNTVYLQMNSLKPEDTAVYYC*AAEVYTYYWYSYYY*WGQGTQVTVSS
D08	QVQLVESGGGLVQAGGSLRLSCAAS*GSTFGTYA*MGWYRQAPGKERELVA*AISGGGNT*NYADSVKGRFTISRDNAKNTVYLQMNSLKPEDTAVYYCAADWYTYTWGFGYSIYYYWGQGTQVTVSS
F10’	QVQLVESGGGLVQAGGSLRLSCAAS*GSTFWWYA*MGWYRQAPGKERELVA*TISRGGST*NYADSVKGRFTISRDNAKNTVYLQMNSLKPEDTAVYYC*ARKTRDIRLDY*WGQGTQVTVSS
G01’	QVQLVESGGGLVQAGGSLRLSCAAS*GITFDRYV*MGWYRQAPGKEREFVA*VISRGGRT*YYADSVKGRFTISRDNAKNTVYLQMNSLKPEDTAVYYC*AASVLLYWYWGEDDY*WGQGTQVTVSS
B10’	QVQLVESGGGLVQAGGSLRLSCAAS*GFIFYYNA*MGWYRQAPGKERELVA*AITWGGGST*YYADSVKGRFTISRDNAKNTVYLQMNSLKPEDTAVYYC*NVLFIAQGSGWTYDY*WGQGTQVTVSS
F11’	QVQLVESGGGLVQAGGSLRLSCAAS*GSTFHHYA*MGWYRQAPGKERELVA*AITTSGGRT*YYADSVKGRFTISRDNAKNTVYLQMNSLKPEDTAVYYC*AADRFFYRGGYYY*WGQGTQVTVSS
D09	QVQLVESGGGLVQAGGSLRLSCAAS*GRTFHWNA*MGWYRQAPGKERELVA*DITSGGST*YYADSVKGRFTISRDNAKNTVYLQMNSLKPEDTAVYYC*AADWFIWWRDDYYAGYDLYDY*WGQGTQVTVSS

F02	QVQLVESGGGLVQAGGSLRLSCAAS*GRIFYSNA*MGWYRQAPGKERELVA*AITFSGAST*YYADSVKGRFTISRDNAKNTVYLQMNSLKPEDTAVYYC*AAEHYNWVSSYRYYY*WGQGTQVTVSS
F01	QVQLVESGGGLVQAGGSLRLSCAAS*GFIFDSYA*MGWYRQAPGKERELVA*AITSSGGT*TYYADSVKGRFTISRDNAKNTVYLQMNSLKPEDTAVYYC*AVRSTFRWYGYYY*WGQGTQVTVSS
D06	QVQLVESGGGLVQAGGSLRLSCAAS*GRIFGYYA*MGWYRQAPGKERELVA*VIRGGVST*NYADSVKGRFTISRDNAKNTVYLQMNSLKPEDTAVYYC*NARRYWAFNAYSKYDY*WGQGTQVTVSS
F09’	QVQLVESGGGLVQAGGSLRLSCAAS*GGTFVRYA*MGWYRQAPGKERELVA*AISSRGDRT*YYADSVKGRFTISRDNAKNTVYLQMNSLKPEDTAVYYC*NTVYYTDSEYDS*WGQGTQVTVSS
G04	QVQLVESGGGLVQAGGSLRLSCAAS*GRIFTGNA*MGWYRQAPGKEREFVA*AISNSGGST*YYADSVKGRFTISRDNAKNTVYLQMNSLKPEDTAVYYC*AASYRVKWKYNY*WGQGTQVTVSS
E06’	QVQLVESGGGLVQAGGSLRLSCAAS*GGTIAYNA*MGWYRQAPGKERELVA*AISSSGGRT*YYADSVKGRFTISRDNAKNTVYLQMNSLKPEDTAVYYC*ATPVTNGFDY*WGQGTQVTVSS
H06	QVQLVESGGGLVQAGGSLRLSCAAS*GSIFYTYA*MGWYRQAPGKERELVA*AITSTGART*YYADSVKGRFTISRDNAKNTVYLQMNSLKPEDTAVYYC*NALSRAGALKYGGPNDY*WGQGTQVTVSS
F10	QVQLVESGGGLVQAGGSLRLSCAAS*GSTFLLYA*MGWYRQAPGKERELVA*AISWSGSRT*YYADSVKGRFTISRDNAKNTVYLQMNSLKPEDTAVYYC*AARSGFAGYGY*WGQGTQVTVSS
H11	QVQLVESGGGLVQAGGSLRLSCAAS*GSTFRYYA*MGWYRQAPGKERELVA*AITRSGAST*YYADSVKGRFTISRDNAKNTVYLQMNSLKPEDTAVYYC*AARSKWNYGRYEY*WGQGTQVTVSS
B01	QVQLVESGGGLVQAGGSLRLSCAAS*GSTFTNYA*MGWYRQAPGKERELVA*AISNNGGRT*YYADSVKGRFTISRDNAKNTVYLQMNSLKPEDTAVYYC*NARIYQGVYVRWYGY*WGQGTQVTVSS
E04’	QVQLVESGGGLVQAGGSLRLSCAAS*GFTFGSYA*MGWYRQAPGKERELVA*AITISGSST*YYADSVKGRFTISRDNAKNTVYLQMNSLKPEDTAVYYC*NAYWGRGYKTEYYY*WGQGTQVTVSS
G02’	QVQLVESGGGLVQAGGSLRLSCAAS*GRIFSRNA*MGWYRQAPGKERELVA*AITQSGGST*YYADSVKGRFTISRDNAKNTVYLQMNSLKPEDTAVYYC*AAVKYWEYDY*WGQGTQVTVSS
H01’	QVQLVESGGGLVQAGGSLRLSCAAS*GRIFGRNT*MGWYRQAPGKERELVA*AIRSGGST*SYADSVKGRFTISRDNAKNTVYLQMNSLKPEDTAVYYC*NVKSYRYLKDGTFTRKYDY*WGQGTQVTVSS
F04’	QVQLVESGGGLVQAGGSLRLSCAAS*GSIFRYYA*MGWYRQAPGKERELVA*AINSRGTST*YYADSVKGRFTISRDNAKNTVYLQMNSLKPEDTAVYYC*NKLVYRYGSYLEMDY*WGQGTQVTVSS
E10’	QVQLVESGGGLVQAGGSLRLSCAAS*GRIFSHYA*MGWYRQAPGKEREFVA*AISADGGS*TYYADSVKGRFTISRDNAKNTVYLQMNSLKPEDTAVYYC*AARKYYRTNGY*WGQGTQVTVSS

F07’	QVQLVESGGGLVQAGGSLRLSCAASGFTFNRYAMGWYRQAPGKERELVAAISGSGASTYYADSVKGRFTISRDNAKNTVYLQMNSLKPEDTAVYYCATRYSRSYRSRDYYYWGQGTQVTVSS
G09’	QVQLVESGGGLVQAGGSLRLSCAASGITFQRYAMGWYRQAPGKERELVASISRSGGSTYYADSVKGRFTISRDNAKNTVYLQMNSLKPEDTAVYYCAARYIVRGGYWGQGTQVTVSS
E05’	QVQLVESGGGLVQAGGSLRLSCAASGYIFVKYAMGWYRQAPGKERELVAAISRSGVRTYYADSVKGRFTISRDNAKNTVYLQMNSLKPEDTAVYYCNAYFYANDYWGQGTQVTVSS
F02’	QVQLVESGGGLVQAGGSLRLSCAASGSTFSRNTMGWYRQAPGKERELVAAISKSGGRTYYADSVKGRFTISRDNAKNTVYLQMNSLKPEDTAVYYCNAAVYAYASDYWGQGTQVTVSS
D06 G26D^{27}	QVQLVESGGGLVQAGGSLRLSCAAS**D**RIFGYYAMGWYRQAPGKERELVAVIRGGVSTNYADSVKGRFTISRDNAKNTVYLQMNSLKPEDTAVYYCNARRYWAFNAYSKYDYWGQGTQVTVSS
D06 T57D^{65}	QVQLVESGGGLVQAGGSLRLSCAASGRIFGYYAMGWYRQAPGKERELVAVIRGGVS**D**NYADSVKGRFTISRDNAKNTVYLQMNSLKPEDTAVYYCNARRYWAFNAYSKYDYWGQGTQVTVSS
D06 G26D^{27} T57D^{65}	QVQLVESGGGLVQAGGSLRLSCAAS**D**RIFGYYAMGWYRQAPGKERELVAVIRGGVS**D**NYADSVKGRFTISRDNAKNTVYLQMNSLKPEDTAVYYCNARRYWAFNAYSKYDYWGQGTQVTVSS
D06 Y32G^{37}	QVQLVESGGGLVQAGGSLRLSCAASGRIFGY**G**AMGWYRQAPGKERELVAVIRGGVSTNYADSVKGRFTISRDNAKNTVYLQMNSLKPEDTAVYYCNARRYWAFNAYSKYDYWGQGTQVTVSS
D06 R52V^{57}	QVQLVESGGGLVQAGGSLRLSCAASGRIFGYYAMGWYRQAPGKERELVAVI**V**GGVSTNYADSVKGRFTISRDNAKNTVYLQMNSLKPEDTAVYYCNARRYWAFNAYSKYDYWGQGTQVTVSS
D06 Y32G^{37} R52V^{57}	QVQLVESGGGLVQAGGSLRLSCAASGRIFGY**G**AMGWYRQAPGKERELVAVI**V**GGVSTNYADSVKGRFTISRDNAKNTVYLQMNSLKPEDTAVYYCNARRYWAFNAYSKYDYWGQGTQVTVSS
D06 Y100-^{109}	QVQLVESGGGLVQAGGSLRLSCAASGRIFGYYAMGWYRQAPGKERELVAVIRGGVSTNYADSVKGRFTISRDNAKNTVYLQMNSLKPEDTAVYYCNARR**-**WAFNAYSKYDYWGQGTQVTVSS
D06 T57D^{65} Y100-^{109}	QVQLVESGGGLVQAGGSLRLSCAASGRIFGYYAMGWYRQAPGKERELVAVIRGGVS**D**NYADSVKGRFTISRDNAKNTVYLQMNSLKPEDTAVYYCNARR**-**WAFNAYSKYDYWGQGTQVTVSS
D06 R98D^{107}	QVQLVESGGGLVQAGGSLRLSCAASGRIFGYYAMGWYRQAPGKERELVAVIRGGVSTNYADSVKGRFTISRDNAKNTVYLQMNSLKPEDTAVYYCNA**D**RYWAFNAYSKYDYWGQGTQVTVSS
D06 R99H^{108}	QVQLVESGGGLVQAGGSLRLSCAASGRIFGYYAMGWYRQAPGKERELVAVIRGGVSTNYADSVKGRFTISRDNAKNTVYLQMNSLKPEDTAVYYCNAR**H**YWAFNAYSKYDYWGQGTQVTVSS
D06 R98D^{107} R99H^{108}	QVQLVESGGGLVQAGGSLRLSCAASGRIFGYYAMGWYRQAPGKERELVAVIRGGVSTNYADSVKGRFTISRDNAKNTVYLQMNSLKPEDTAVYYCNARRYWAFNAYSKYDYWGQGTQVTVSS

D06<br>A97H^{106}	QVQLVESGGGLVQAGGSLRLSCAAS*GRIFGYYA*MGWYRQAPGKERELVA*VIRGGVST*NYADSVKGRFTISRDNAKNTVYLQMNSLKPEDTAVYYC*N**H**RRYWAFNAYSKYDY*WGQGTQVTVSS
D06<br>N96W^{106}	QVQLVESGGGLVQAGGSLRLSCAAS*GRIFGYYA*MGWYRQAPGKERELVA*VIRGGVST*NYADSVKGRFTISRDNAKNTVYLQMNSLKPEDTAVYYC**W***ARRYWAFNAYSKYDY*WGQGTQVTVSS
D06<br>G54F^{59}<br>R98D^{107}	QVQLVESGGGLVQAGGSLRLSCAAS*GRIFGYYA*MGWYRQAPGKERELVA*VIRG**F**VST*NYADSVKGRFTISRDNAKNTVYLQMNSLKPEDTAVYYC*NA**D**RYWAFNAYSKYDY*WGQGTQVTVSS
D06<br>G26D^{27}<br>R98D^{107}	QVQLVESGGGLVQAGGSLRLSCAAS**D***RIFGYYA*MGWYRQAPGKERELVA*VIRGGVST*NYADSVKGRFTISRDNAKNTVYLQMNSLKPEDTAVYYC*NA**D**RYWAFNAYSKYDY*WGQGTQVTVSS
D06<br>G54F^{59}	QVQLVESGGGLVQAGGSLRLSCAAS*GRIFGYYA*MGWYRQAPGKERELVA*VIRG**F**VST*NYADSVKGRFTISRDNAKNTVYLQMNSLKPEDTAVYYC*NARRYWAFNAYSKYDY*WGQGTQVTVSS
D06<br>F29R^{30}	QVQLVESGGGLVQAGGSLRLSCAAS*GRI**R**GYYA*MGWYRQAPGKERELVA*VIRGGVST*NYADSVKGRFTISRDNAKNTVYLQMNSLKPEDTAVYYC*NARRYWAFNAYSKYDY*WGQGTQVTVSS
D06<br>F29R^{30}<br>T57D^{65}	QVQLVESGGGLVQAGGSLRLSCAAS*GRI**R**GYYA*MGWYRQAPGKERELVA*VIRGGVS***D**NYADSVKGRFTISRDNAKNTVYLQMNSLKPEDTAVYYC*NARRYWAFNAYSKYDY*WGQGTQVTVSS
D06<br>G26D^{27}<br>A97H^{106}	QVQLVESGGGLVQAGGSLRLSCAAS**D***RIFGYYA*MGWYRQAPGKERELVA*VIRGGVST*NYADSVKGRFTISRDNAKNTVYLQMNSLKPEDTAVYYC*N**H**RRYWAFNAYSKYDY*WGQGTQVTVSS
D06<br>G26D^{27}<br>Y32G^{37}	QVQLVESGGGLVQAGGSLRLSCAAS**D***RIFGY**G**A*MGWYRQAPGKERELVA*VIRGGVST*NYADSVKGRFTISRDNAKNTVYLQMNSLKPEDTAVYYC*NARRYWAFNAYSKYDY*WGQGTQVTVSS
D06<br>R52V^{57}<br>T57D^{65}	QVQLVESGGGLVQAGGSLRLSCAAS*GRIFGYYA*MGWYRQAPGKERELVA*VI**V**GGVS***D**NYADSVKGRFTISRDNAKNTVYLQMNSLKPEDTAVYYC*NARRYWAFNAYSKYDY*WGQGTQVTVSS
E10’<br>G26D^{27}	QVQLVESGGGLVQAGGSLRLSCAAS**D***RIFSHYA*MGWYRQAPGKEREFVA*AISADGGS*TYYADSVKGRFTISRDNAKNTVYLQMNSLKPEDTAVYYC*AARKYYRTNGY*WGQGTQVTVSS
E10’<br>F29R^{30}	QVQLVESGGGLVQAGGSLRLSCAAS*GRI**R**SHYA*MGWYRQAPGKEREFVA*AISADGGS*TYYADSVKGRFTISRDNAKNTVYLQMNSLKPEDTAVYYC*AARKYYRTNGY*WGQGTQVTVSS
E10’<br>G26D^{27}<br>F29R^{30}	QVQLVESGGGLVQAGGSLRLSCAAS**D***RI**R**SHYA*MGWYRQAPGKEREFVA*AISADGGS*TYYADSVKGRFTISRDNAKNTVYLQMNSLKPEDTAVYYC*AARKYYRTNGY*WGQGTQVTVSS
E10’<br>Y32G^{37}	QVQLVESGGGLVQAGGSLRLSCAAS*GRIFSH**G**A*MGWYRQAPGKEREFVA*AISADGGS*TYYADSVKGRFTISRDNAKNTVYLQMNSLKPEDTAVYYC*AARKYYRTNGY*WGQGTQVTVSS
E10’<br>T58D^{65}	QVQLVESGGGLVQAGGSLRLSCAAS*GRIFSHYA*MGWYRQAPGKEREFVA*AISADGGS***D**YYADSVKGRFTISRDNAKNTVYLQMNSLKPEDTAVYYC*AARKYYRTNGY*WGQGTQVTVSS

E10'<br>Y32G^{37}<br>T58D^{65}	QVQLVESGGGLVQAGGSLRLSCAASGRIFSHYAMGWYRQAPGKEREFVAAISA<br>DGGS**D**YYADSVKGRFTISRDNAKNTVYLQMNSLKPEDTAVYYCAARKYYRTNG<br>YWGQGTQVTVSS
E10'<br>S52V^{57}	QVQLVESGGGLVQAGGSLRLSCAASGRIFSHYAMGWYRQAPGKEREFVAAI**V**A<br>DGGSTYYADSVKGRFTISRDNAKNTVYLQMNSLKPEDTAVYYCAARKYYRTNG<br>YWGQGTQVTVSS
E10'<br>R99D^{107}	QVQLVESGGGLVQAGGSLRLSCAASGRIFSHYAMGWYRQAPGKEREFVAAISA<br>DGGSTYYADSVKGRFTISRDNAKNTVYLQMNSLKPEDTAVYYCAARKYYRTNG<br>YWGQGTQVTVSS
E10'<br>S52V^{57}<br>R99D^{107}	QVQLVESGGGLVQAGGSLRLSCAASGRIFSHYAMGWYRQAPGKEREFVAAISA<br>DGGSTYYADSVKGRFTISRDNAKNTVYLQMNSLKPEDTAVYYCAARKYYRTNG<br>YWGQGTQVTVSS
E10'<br>G55H^{62}	QVQLVESGGGLVQAGGSLRLSCAASGRIFSHYAMGWYRQAPGKEREFVAAISA<br>D**H**GSTYYADSVKGRFTISRDNAKNTVYLQMNSLKPEDTAVYYCAARKYYRTNG<br>YWGQGTQVTVSS
E10'<br>G55H^{62}<br>T58D^{65}	QVQLVESGGGLVQAGGSLRLSCAASGRIFSHYAMGWYRQAPGKEREFVAAISA<br>D**H**GSTYYADSVKGRFTISRDNAKNTVYLQMNSLKPEDTAVYYCAARKYYRTNG<br>YWGQGTQVTVSS
E10'<br>Y102E^{110}	QVQLVESGGGLVQAGGSLRLSCAASGRIFSHYAMGWYRQAPGKEREFVAAISA<br>DGGSTYYADSVKGRFTISRDNAKNTVYLQMNSLKPEDTAVYYCAARKY**E**RTNG<br>YWGQGTQVTVSS
E10'<br>R99D^{107}<br>Y102E^{110}	QVQLVESGGGLVQAGGSLRLSCAASGRIFSHYAMGWYRQAPGKEREFVAAISA<br>DGGSTYYADSVKGRFTISRDNAKNTVYLQMNSLKPEDTAVYYCAA**D**KY**E**RTNG<br>YWGQGTQVTVSS
E10'<br>A97W^{105}	QVQLVESGGGLVQAGGSLRLSCAASGRIFSHYAMGWYRQAPGKEREFVAAISA<br>DGGSTYYADSVKGRFTISRDNAKNTVYLQMNSLKPEDTAVYYC**W**ARKYYRTNG<br>YWGQGTQVTVSS
E10'<br>G26D^{27}<br>A97W^{105}	QVQLVESGGGLVQAGGSLRLSCAAS**D**RIFSHYAMGWYRQAPGKEREFVAAISA<br>DGGSTYYADSVKGRFTISRDNAKNTVYLQMNSLKPEDTAVYYC**W**ARKYYRTNG<br>YWGQGTQVTVSS
E10'<br>A98H^{106}	QVQLVESGGGLVQAGGSLRLSCAASGRIFSHYAMGWYRQAPGKEREFVAAISA<br>DGGSTYYADSVKGRFTISRDNAKNTVYLQMNSLKPEDTAVYYCA**H**RKYYRTNG<br>YWGQGTQVTVSS
AT118<br>h32	EVQLVESGGGLVQPGGSLRLSCAASGYIYRRYRMGWYRQAPGKGREFVAAISG<br>GSSTNYADSVKGRFTISRDNSKNTVYLQMNSLRAEDTAVYYCAAYRIVSDPRV<br>YWGQGTQVTVSS
AT118<br>h32<br>G26D^{27}	EVQLVESGGGLVQPGGSLRLSCAAS**D**YIYRRYRMGWYRQAPGKGREFVAAISG<br>GSSTNYADSVKGRFTISRDNSKNTVYLQMNSLRAEDTAVYYCAAYRIVSDPRV<br>YWGQGTQVTVSS
AT118<br>h32<br>Y29R^{30}	EVQLVESGGGLVQPGGSLRLSCAASGYI**R**RRYRMGWYRQAPGKGREFVAAISG<br>GSSTNYADSVKGRFTISRDNSKNTVYLQMNSLRAEDTAVYYCAAYRIVSDPRV<br>YWGQGTQVTVSS
AT118<br>h32<br>R31D^{36}	EVQLVESGGGLVQPGGSLRLSCAASGYIYR**D**YRMGWYRQAPGKGREFVAAISG<br>GSSTNYADSVKGRFTISRDNSKNTVYLQMNSLRAEDTAVYYCAAYRIVSDPRV<br>YWGQGTQVTVSS

AT118 h32 Y32G^{37}	EVQLVESGGGLVQPGGSLRLSCAASGYIYRR**G**RMGWYRQAPGKGREFVAAISGGSSTNYADSVKGRFTISRDNSKNTVYLQMNSLRAEDTAVYYCAAYRIVSDPRVYWGQGTQVTVSS
AT118 h32 S52V^{57}	EVQLVESGGGLVQPGGSLRLSCAASGYIYRRYRMGWYRQAPGKGREFVAAI**V**GGSSTNYADSVKGRFTISRDNSKNTVYLQMNSLRAEDTAVYYCAAYRIVSDPRVYWGQGTQVTVSS
AT118 h32 G54F^{59}	EVQLVESGGGLVQPGGSLRLSCAASGYIYRRYRMGWYRQAPGKGREFVAAISG**F**SSTNYADSVKGRFTISRDNSKNTVYLQMNSLRAEDTAVYYCAAYRIVSDPRVYWGQGTQVTVSS
AT118 h32 T57D^{65}	EVQLVESGGGLVQPGGSLRLSCAASGYIYRRYRMGWYRQAPGKGREFVAAISGGSS**D**NYADSVKGRFTISRDNSKNTVYLQMNSLRAEDTAVYYCAAYRIVSDPRVYWGQGTQVTVSS
AT118 h32 T57I^{65}	EVQLVESGGGLVQPGGSLRLSCAASGYIYRRYRMGWYRQAPGKGREFVAAISGGSS**I**NYADSVKGRFTISRDNSKNTVYLQMNSLRAEDTAVYYCAAYRIVSDPRVYWGQGTQVTVSS
AT118 h32 A96W^{105}	EVQLVESGGGLVQPGGSLRLSCAASGYIYRRYRMGWYRQAPGKGREFVAAISGGSSTNYADSVKGRFTISRDNSKNTVYLQMNSLRAEDTAVYYC**W**AYRIVSDPRVYWGQGTQVTVSS
AT118 h32 A97H^{106}	EVQLVESGGGLVQPGGSLRLSCAASGYIYRRYRMGWYRQAPGKGREFVAAISGGSSTNYADSVKGRFTISRDNSKNTVYLQMNSLRAEDTAVYYCA**H**YRIVSDPRVYWGQGTQVTVSS
AT118 h32 Y98D^{107}	EVQLVESGGGLVQPGGSLRLSCAASGYIYRRYRMGWYRQAPGKGREFVAAISGGSSTNYADSVKGRFTISRDNSKNTVYLQMNSLRAEDTAVYYCAA**D**RIVSDPRVYWGQGTQVTVSS
AT118 h32 R99D^{108}	EVQLVESGGGLVQPGGSLRLSCAASGYIYRRYRMGWYRQAPGKGREFVAAISGGSSTNYADSVKGRFTISRDNSKNTVYLQMNSLRAEDTAVYYCAAY**D**IVSDPRVYWGQGTQVTVSS
AT118 h32 G26D^{27} T57I^{65}	EVQLVESGGGLVQPGGSLRLSCAAS**D**YIYRRYRMGWYRQAPGKGREFVAAISGGSS**I**NYADSVKGRFTISRDNSKNTVYLQMNSLRAEDTAVYYCAAYRIVSDPRVYWGQGTQVTVSS
Cablivi (12A2H1)	EVQLVESGGGLVQPGGSLRLSCAASGRTFSYNPMGWFRQAPGKGRELVAAISRTGGSTYYPDSVEGRFTISRDNAKRMVYLQMNSLRAEDTAVYYCAAAGVRAEDGRVRTLPSEYTFWGQGTQVTVSS

Supplementary Table 2. Orthorep Substitutions

Clone	Total # substitutions detected	# substitutions computationally predicted to decrease polyreactivity one-hot	# substitutions computationally predicted to decrease polyreactivity k-mer
E05’	240	233	234
F02’	49	33	32
G09’	62	43	47
F07’	14	13	13

Supplementary Table 3. Table of nanobody rescue mutation predictions with models trained on deeper FACS sorting experiments

Name	Logistic regression onehot CDRS original	Logistic regression 3mer CDRS original	Logistic regression onehot CDRS deep	Logistic regression 3mer CDRS deep	CNN CDRS deep	RNN CDRS deep
AT118 h32 G26D^{27}	1.42	-1.11	-0.70	-0.77	0.60	0.51
AT118 h32 R31D^{36}	0.94	0.33	0.09	0.71	0.69	0.27
AT118 h32 Y29R^{30}	0.96	-1.29	-1.15	-1.41	0.52	0.55
AT118 h32 Y32G^{37}	0.81	-1.33	0.40	-1.19	0.47	0.55
AT118 h32 G54F^{59}	1.25	-0.73	-1.08	-0.53	0.48	0.57
AT118 h32 S52V^{57}	1.19	-0.47	-0.57	-0.33	0.48	0.53
AT118 h32 T57I^{65}	1.20	-0.71	-1.04	-0.27	0.40	0.54
AT118 h32 A96W^{105}	0.95	-0.75	-1.16	-0.72	0.57	0.61
AT118 h32 A97H^{106}	0.91	-0.28	-0.70	-0.58	0.48	0.36
AT118 h32 R99D^{108}	0.78	-0.69	-0.15	-0.07	0.61	0.73
AT118 h32 Y98D^{107}	1.03	0.22	-0.33	-0.11	0.61	0.65
AT118 h32	-0.33	-1.11	-1.11	-0.87	0.48	0.56
E10’ F29R^{30}	-0.52	-2.44	-1.67	-2.16	0.25	0.13
E10’ G26D^{27}	-0.08	-2.73	-1.44	-1.18	0.33	0.17
E10’ G26D^{27} F29R^{30}	1.22	-2.44	-1.27	-1.84	0.39	0.14
E10’ G26D^{27} A97W^{105}	1.20	-2.02	-1.49	-0.84	0.40	0.17
E10’ Y32G^{37}	-0.69	-2.40	-0.33	-1.02	0.23	0.17
E10’ Y32G^{37} T58D^{65}	1.46	-1.82	-0.33	-0.32	0.26	0.17
E10’ G55H^{62}	-0.10	-2.67	-2.16	-1.25	0.32	0.17
E10’ G55H^{62} T58D^{65}	2.04	-2.09	-2.16	-0.56	0.31	0.18
E10’ S52V^{57}	-0.31	-1.67	-1.30	-1.23	0.28	0.15
E10’ S52V^{57} R99D^{107}	1.25	0.22	-0.21	0.06	0.67	0.17

E10’ T58D^{65}	0.33	-2.15	-1.84	-0.80	0.23	0.16
E10’ A97W^{105}	-0.54	-2.02	-1.89	-1.15	0.29	0.18
E10’ A98H^{106}	-0.59	-1.84	-1.44	-0.90	0.25	0.18
E10’ R99D^{107}	-0.27	-0.84	-0.74	-0.20	0.55	0.17
E10’ R99D^{107} Y102E^{110}	0.80	-0.07	-0.12	1.04	0.73	0.24
E10’ Y102E^{110}	-0.75	-1.96	-1.21	-0.26	0.38	0.17
E10’	-1.82	-2.73	-1.84	-1.50	0.21	0.15
D06 F29R^{30}	1.49	-1.34	-0.71	-1.87	0.27	0.18
D06 F29R^{30} T57D^{65}	3.64	-0.85	-0.71	-1.70	0.43	0.27
D06 G26D^{27}	1.93	-1.07	-0.47	-0.93	0.35	0.24
D06 G26D^{27} Y32G^{37}	3.07	-0.61	1.04	-0.60	0.37	0.20
D06 G26D^{27} T57D^{65}	4.08	-0.58	-0.48	-0.76	0.41	0.16
D06 G26D^{27} A97H^{106}	3.17	-0.46	-0.07	-1.00	0.38	0.24
D06 G26D^{27} R98D^{107}	3.49	0.68	0.62	0.15	0.61	0.36
D06 Y32G^{37}	1.32	-0.61	0.63	-0.88	0.29	0.19
D06 Y32G^{37} R52V^{57}	2.83	0.00	1.47	-0.05	0.65	0.30
D06 G54F^{59}	1.77	-0.55	-0.85	-0.69	0.27	0.24
D06 G54F^{59} R98D^{107}	3.32	1.19	0.24	0.39	0.71	0.30
D06 R52V^{57}	1.70	-0.46	-0.04	-0.38	0.59	0.28
D06 R52V^{57} T57D^{65}	3.84	0.03	-0.04	-0.21	0.66	0.19
D06 T57D^{65}	2.34	-0.58	-0.88	-1.04	0.40	0.24
D06 T57D^{65} Y100-^{109}	3.13	0.14	-0.54	-1.14	0.46	0.25
D06 A97H^{106}	1.42	-0.46	-0.48	-1.28	0.28	0.24
D06 N96W^{106}	1.36	-0.85	-1.02	-1.17	0.28	0.24
D06 R98D^{107}	1.74	0.68	0.22	-0.13	0.70	0.33
D06 R99H^{108}	2.81	1.38	0.80	0.33	0.78	0.61
D06 R98D$_{107}$ R99H^{108}	1.26	0.14	-0.29	-0.68	0.34	0.32
D06 Y100-^{109}	0.98	-0.37	-0.54	-1.32	0.29	0.30
D06	0.19	-1.07	-0.88	-1.21	0.26	0.26

Supplementary Table 4. Crystallography data collection and refinement statistics

	AT118i4h32	AT118i4h32 G26D^{27} T57I^{65}
**PDB ID**	7T83	7T84
**Data collection**		
Space group	$P2_1$	$P2_1$
Cell dimensions		
a, *b*, *c* (Å)	80.1, 85.4, 84.4	78.7, 89.2, 83.1
α, β, γ (°)	90, 108.6, 90	90, 110.1, 90
Resolution (Å)	31.5 – 2.1 (2.2 – 2.1) *	46.3 – 1.6 (1.7-1.6)
R_{merge} (%)	6.8 (174.3)	8.9 (174.9)
$CC_{1/2}$ (%)	99.8 (67.1)	99.9 (53.6)
I / □*I*	12.1 (0.84)	13.4 (1.4)
Completeness (%)	98.9 (97.2)	98.6 (97.4)
Redundancy	6.8 (6.7)	6.8 (7.1)
**Refinement**		
Resolution (Å)	31.5 – 2.1	46.3 – 1.6
No. reflections	62627	139941
R_{work} / R_{free} (%)	20.8/25.7	16.1 / 18.9
No. atoms	7428	8651
Protein	7305	7691
Ligand	52	139
Water	71	821
B-factors	86.3	32.3
Protein	86.4	31.3
Ligand	107.3	41.0
Water	68.7	39.9
R.m.s. deviations		
Bond lengths (Å)	0.003	0.02
Bond angles (°)	0.9	1.62

*Values in parentheses are for highest-resolution shell

Supplementary Table 5. Melting temperatures of AT118i4h32 variants

	T_m (°C)
AT118i4h32	64.0 ± 0.05
AT118i4h32 G26D[27]	66.3 ± 0.05
AT118i4h32 T57I[65]	62.8 ± 0.31
AT118i4h32 G26D[27] T57I[65]	65.7 ± 0.06

Effect of G26D[27] and T57I[65] substitutions on melting temperature (T_m, °C) of AT118i4h32. Error represents standard error of the mean determined from three independent experiments performed in technical triplicates.

Supplementary Table 6. Table of nanobody K_i values

	[3H]-Olmesartan / Membrane AT1R
Losartan	-7.34 ± 0.07<br>(45.7 nM)
AT118i4h32	-7.14 ± 0.08<br>(72.4 nM)
AT118i4h32 G26D^{27}	-7.15 ± 0.12<br>(70.8 nM)
AT118i4h32 T57I^{65}	-7.19 ± 0.13<br>(64.6 nM)
AT118i4h32 G26D^{27} T57I^{65}	-7.10 ± 0.14<br>(79.4 nM)

Effects of G26D^{27} and T57I^{65} substitutions on ligand binding. Log Ki values (Ki values shown in parentheses) were determined using the Kd value of [3H]-Olmesartan (Kd 0.29 +/- 0.03 nM). Error represents standard error of the mean from three independent experiments.

Supplementary Table 7. Table of nanobody polyreactivity algorithm predictions for heavy chain antibodies currently in clinical development.

Therapeutic	Bispecific (Yes/No)	Clinical trial stage	Heavy chain number	Target	CDR3 sequence	P_i value	Hydro-phobicity	One hot model polyreactivity prediction
Caplacizumab	No	Approved	1	VWF	AAAGVRAEDG RVRTLPSEYTF	9.96	-0.52	0.16
Envafolimab	No	Preregistration	1	PDL1	AADS	10.05	-0.55	1.08
Letolizumab	No	Phase-I/II	1	CD40LG	VKVGKDAKSDY	4.44	-0.44	-0.45
Rimteravimab	No	Phase-I/II	1	CoV2 RBD	AFITTESDYDL GRRY	4.05	-0.24	3.43
Gefurulimab	Yes	Phase-I	1	C5	TIGGSLSR	9.31	-0.45	0.59
			2	ALB	AARQGQYIYS SMRSDSYDY	5.36	-0.97	2.53
Isecarosmab	Yes	Phase-I	1	ADAMTSL5	AADLDPNRIF SREEYAY	6.28	-0.78	1.02
			2	ALB	TIGGSLSR	5.84	0.09	1.61
Vobarilizumab	Yes	Phase-II (Disc.)	1	IL6R	AFITTESDYDL GRRY	6.12	0.03	1.29
			2	ALB	TIGGSLSR	5.84	0.09	1.61
Erfonrilimab	Yes	Phase-II	1	CD274	AADVIPTETCL GGSWSGPFGY	8.03	-0.29	2.69
			2	CTLA4	AADVIPTETCL GGSWSGPFGY	4.05	0.39	3.51
Gontivimab	Yes	Phase-II (Disc.)	1	RSV gpF	GAGTPLNPGA YIYDWSYDY	4.05	-0.35	2
			2	RSV gpF	GAGTPLNPGA YIYDWSYDY	4.05	-0.35	2
Ozoralizumab	Yes	Preregistration	1	TNFA	ARSPSGFN	4.37	-0.34	-0.42
			2	ALB	TIGGSLSR	5.84	0.09	1.61
