# Supplementary material for: An in silico method to assess antibody fragment polyreactivity
Source: Nat Commun. 2022 Dec 7;13:7554. doi: 10.1038/s41467-022-35276-4 (PMC9729196; doi:10.1038/s41467-022-35276-4)
Supplement: Supplementary file 2 — Reporting Summary [file 41467_2022_35276_MOESM2_ESM.pdf]

Corresponding author(s): Debora Marks and Andrew Kruse

Last updated by author(s): Nov 14, 2022

Reporting Summary

Nature Portfolio wishes to improve the reproducibility of the work that we publish. This form provides structure for consistency and transparency in reporting. For further information on Nature Portfolio policies, see our Editorial Policies and the Editorial Policy Checklist.

Statistics

For all statistical analyses, confirm that the following items are present in the figure legend, table legend, main text, or Methods section.

n/a	Confirmed	
☐	☒	The exact sample size (*n*) for each experimental group/condition, given as a discrete number and unit of measurement
☐	☒	A statement on whether measurements were taken from distinct samples or whether the same sample was measured repeatedly
☐	☒	The statistical test(s) used AND whether they are one- or two-sided<br>*Only common tests should be described solely by name; describe more complex techniques in the Methods section.*
☒	☐	A description of all covariates tested
☒	☐	A description of any assumptions or corrections, such as tests of normality and adjustment for multiple comparisons
☐	☒	A full description of the statistical parameters including central tendency (e.g. means) or other basic estimates (e.g. regression coefficient) AND variation (e.g. standard deviation) or associated estimates of uncertainty (e.g. confidence intervals)
☐	☒	For null hypothesis testing, the test statistic (e.g. *F*, *t*, *r*) with confidence intervals, effect sizes, degrees of freedom and *P* value noted<br>*Give P values as exact values whenever suitable.*
☒	☐	For Bayesian analysis, information on the choice of priors and Markov chain Monte Carlo settings
☒	☐	For hierarchical and complex designs, identification of the appropriate level for tests and full reporting of outcomes
☒	☐	Estimates of effect sizes (e.g. Cohen's *d*, Pearson's *r*), indicating how they were calculated

Our web collection on statistics for biologists contains articles on many of the points above.

Software and code

Policy information about availability of computer code

Data collection	Only commercial software was used. Software used for X-ray data processing is described in the methods section with references to relevant literature. Software was supported by the SBGrid consortium. FACS data were collected using the SONY SH800 (version 2.1.6) and BD Accuri C6 flow cytometry software (version 1.0.264.21). Flow cytometry data were plotted using FlowJo (version 10.8.1).
Data analysis	Data from yeast staining experiments, nanobody ELISA assays, AC-SINS assays, radioligand binding and cell-based functional assay experiments were analyzed in GraphPad Prism (version 9.2.0). NGS analysis, sequence processing and model development are described in the methods section. The code for scoring new sequences for polyreactivity, designing rescue mutations, training polyreactivity models, and calculating biochemical properties of a sequence can be found on github: https://github.com/debbiemarkslab/nanobody-polyreactivity, and the webserver is available here: http://18.224.60.30:3000/.

For manuscripts utilizing custom algorithms or software that are central to the research but not yet described in published literature, software must be made available to editors and reviewers. We strongly encourage code deposition in a community repository (e.g. GitHub). See the Nature Portfolio guidelines for submitting code & software for further information.

Data

Policy information about availability of data

All manuscripts must include a data availability statement. This statement should provide the following information, where applicable:

- Accession codes, unique identifiers, or web links for publicly available datasets
- A description of any restrictions on data availability
- For clinical datasets or third party data, please ensure that the statement adheres to our policy

All data generated for the present study are available by request to the corresponding authors. Coordinates and structure factors for the AT118i4h32 structures are deposited in the pDB with accession codes 7T83 and 7T84.

Human research participants

Policy information about studies involving human research participants and Sex and Gender in Research.

Reporting on sex and gender	n/a
Population characteristics	n/a
Recruitment	n/a
Ethics oversight	n/a

Note that full information on the approval of the study protocol must also be provided in the manuscript.

Field-specific reporting

Please select the one below that is the best fit for your research. If you are not sure, read the appropriate sections before making your selection.

☒ Life sciences ☐ Behavioural & social sciences ☐ Ecological, evolutionary & environmental sciences

For a reference copy of the document with all sections, see nature.com/documents/nr-reporting-summary-flat.pdf

Life sciences study design

All studies must disclose on these points even when the disclosure is negative.

Sample size	All experiments were performed in at least biological duplicates and sample sizes for each experiment are indicated in figure legends. We chose sample sizes to ensure reproducibility in accordance to standard operating procedures for each of the experiments.
Data exclusions	No data were excluded from analysis.
Replication	For ELISA and AC-SINS assays, two biological replicate experiments were performed in technical triplicate. For all other experiments, a minimum of three independent experiments were performed with technical triplicates. All replicate attempts were successful.
Randomization	We randomly selected yeast clones from MACS and FACS pools to generate our representative index set of 48 nanobodies that possess varying levels of polyreactivity.
Blinding	To assess computational model performance, only the sequences of the index set of nanobodies (and not their respective polyreactivity measurements) were provided to the computational biologists who designed the algorithm. Then, we correlated the computational predictions to experimental measurements.

Reporting for specific materials, systems and methods

We require information from authors about some types of materials, experimental systems and methods used in many studies. Here, indicate whether each material, system or method listed is relevant to your study. If you are not sure if a list item applies to your research, read the appropriate section before selecting a response.

Materials & experimental systems

n/a	Involved in the study
☐	☒ Antibodies
☐	☒ Eukaryotic cell lines
☒	☐ Palaeontology and archaeology
☒	☐ Animals and other organisms
☒	☐ Clinical data
☒	☐ Dual use research of concern

Methods

n/a	Involved in the study
☒	☐ ChIP-seq
☐	☒ Flow cytometry
☒	☐ MRI-based neuroimaging

Antibodies

Antibodies used

HRP-anti v5 antibody (Abcam cat# ab1325), Alexafluor 647 conjugated anti-v5 antibody (ThermoFisher cat# 451098), Monorab Rabbit Anti-Camelid VHH Antibody (Genescript cat# A01860, Clone 96A3F5), anti-FLAG M1 (ATCC hybridoma line HB9259), anti-HA (Clone 12CA5), Alexafluor-488 conjugated streptavidin (Biolegend cat #405235).

Validation

For each of the primary antibodies used for epitope tags (HA), we tested uninduced cell cultures to validate that no binding signal could be detected unless cells were induced for surface display. All secondary antibodies were validated to bind only in the presence of their respective relevant antigen. In addition, antibodies were used in our labs successfully in unrelated experiments and were validated by the manufacturers according to standard protocols: the HRP-anti V5 antibody (Abcam) was validated via direct Elisa using v5-tagged BSA protein, Alexafluor 647 conjugated anti-v5 antibody (ThermoFisher) was successfully used in 7 publications to date. Monorab Rabbit Anti-Camelid VHH Antibody (Genescript) has been used in 3 publications and has no cross-reactivity with chicken, goat, human, mouse, and rat immunoglobulins. The Alexafluor-488 conjugated streptavidin was validated by the manufacturer using human peripheral blood lymphocytes that were stained with either biotinylated CD3 or biotinylated mouse IgG1 isotype control.

Eukaryotic cell lines

Policy information about cell lines and Sex and Gender in Research

Cell line source(s)

Expi293F (Thermo Fisher Scientific). The derivative tetracycline-inducible Expi293R cell line used fro protein expression was reported in Staus et al, Proc Natl Acad Sci USA 115, 3834-3839 (2018), and the derivative stable cell lines expressing FLAG-human AT1R wild-type was generated in this study. BJ5465 yeast (ATCC), Sf9 insect cells (Expression Systems).

Authentication

No additional authentication of cell lines was performed.

Mycoplasma contamination

All cell lines tested negative for mycoplasma contamination.

Commonly misidentified lines
(See ICLAC register)

None were used.

Flow Cytometry

Plots

Confirm that:

☒ The axis labels state the marker and fluorochrome used (e.g. CD4-FITC).

☒ The axis scales are clearly visible. Include numbers along axes only for bottom left plot of group (a 'group' is an analysis of identical markers).

☒ All plots are contour plots with outliers or pseudocolor plots.

☒ A numerical value for number of cells or percentage (with statistics) is provided.

Methodology

Sample preparation

All sample preparations for flow cytometry are described in the methods section: "Yeast Sorting", "Anti-VHH Antibody Staining", and "Polyspecificity Reagent Analytical Staining."

Instrument

BD Accuri C6 flow cytometer, Sony SH800 flow cytometer

Software

FlowJo 10.8.1, SONY SH800 Software version 2.1.6

Cell population abundance

Flow cytometry in our study was not used to determine relative abundances among populations.

Gating strategy

For all flow cytometry experiments, the gating strategies are shown in the extended data figures. For FCS/SSC gating, the central population was gated. To select for single cells, the population forming a uniform population with a 1:1 ratio between FSC-H and FSC-A was gated.

☒ Tick this box to confirm that a figure exemplifying the gating strategy is provided in the Supplementary Information.
